# Transethosomal Gel for the Topical Delivery of Celecoxib: Formulation and Estimation of Skin Cancer Progression

**DOI:** 10.3390/pharmaceutics15010022

**Published:** 2022-12-21

**Authors:** Ahmed A. H. Abdellatif, Basmah Nasser Aldosari, Amal Al-Subaiyel, Aisha Alhaddad, Waad A. Samman, Nermin E. Eleraky, Marwa G. Elnaggar, Hassan Barakat, Hesham M. Tawfeek

**Affiliations:** 1 Department of Pharmaceutics, College of Pharmacy, Qassim University, Qassim 51452, Saudi Arabia; 2 Department of Pharmaceutics and Pharmaceutical Technology, Faculty of Pharmacy, Al-Azhar University, Assiut 71524, Egypt; 3 Department of Pharmaceutics, College of Pharmacy, King Saud University, Riyadh 11495, Saudi Arabia; 4 Department of Pharmacology, College of Pharmacy, Taibah University, Medina 42353, Saudi Arabia; 5 Department of Pharmacology and Toxicology, College of Pharmacy College, Taibah University, Medina 42353, Saudi Arabia; 6 Department of Pharmaceutics, Faculty of Pharmacy, Assiut University, Assiut 71526, Egypt; 7 Department of Industrial Pharmacy, Faculty of Pharmacy, Assiut University, Assiut 71526, Egypt; 8 Department of Industrial and Physical Pharmacy, Purdue University, 575 Stadium Mall Drive, West Lafayette, IN 47907, USA; 9 Department of Food Science and Human Nutrition, College of Agriculture and Veterinary Medicine, Qassim University, Buraydah 51452, Saudi Arabia; 10 Food Technology Department, Faculty of Agriculture, Benha University, Moshtohor 13736, Egypt

**Keywords:** transethosomal nanovesicles, topical gel delivery, celecoxib, skin cancer progression

## Abstract

The topical delivery of therapeutics is a promising strategy for managing skin conditions. Cyclooxygenase-2 (COX-2) inhibitors showed a possible target for chemoprevention and cancer management. Celecoxib (CXB) is a selective COX-2 inhibitor that impedes cell growth and generates apoptosis in different cell tumors. Herein, an investigation proceeded to explore the usefulness of nano lipid vesicles (transethosomes) (TES) of CXB to permit penetration of considerable quantities of the drug for curing skin cancer. The prepared nanovesicles were distinguished for drug encapsulation efficiency, vesicle size, PDI, surface charge, and morphology. In addition, FT-IR and DSC analyses were also conducted to examine the influence of vesicle components. The optimized formulation was dispersed in various hydrogel bases. Furthermore, *in vitro* CXB release and ex vivo permeability studies were evaluated. A cytotoxicity study proceeded using A431 and BJ1 cell lines. The expression alteration of the cyclin-dependent kinase inhibitor 2A (CDKN2A) gene and DNA damage and fragmentation using qRT-PCR and comet assays were also investigated. Optimized CXB-TES formulation was spherically shaped and displayed a vesicle size of 75.9 ± 11.4 nm, a surface charge of −44.7 ± 1.52 mV, and an entrapment efficiency of 88.8 ± 7.2%. The formulated TES-based hydrogel displayed a sustained *in vitro* CXB release pattern for 24 h with an enhanced flux and permeation across rat skin compared with the control (free drug-loaded hydrogel). Interestingly, CXB-TES hydrogel has a lower cytotoxic effect on normal skin cells compared with TES suspension and CXB powder. Moreover, the level of expression of the CDKN2A gene was significantly (*p* ≤ 0.01, ANOVA/Tukey) decreased in skin tumor cell lines compared with normal skin cell lines, indicating that TES are the suitable carrier for topical delivery of CXB to the cancer cells suppressing their progression. In addition, apoptosis demonstrated by comet and DNA fragmentation assays was evident in skin cancer cells exposed to CXB-loaded TES hydrogel formulation. In conclusion, our results illustrate that CXB-TES-loaded hydrogel could be considered a promising carrier and effective chemotherapeutic agent for the management of skin carcinoma.

## 1. Introduction

One of the most prevalent types of skin cancer is squamous cell carcinoma (SCC), which accounts for 20% of non-melanocytic skin cancers [1]. This type of skin cancer develops in skin areas regularly exposed to UV light, such as the face and arms [2]. The most frequent treatment for SCC is surgical resection and chemotherapy [3]. However, chemotherapy has many disadvantages due to undesired distribution to normal cells [2]. The role of inflammatory cells in the cancer environment and their actions in cell proliferation, migration, and survival have been addressed to target them in treatment. The Cyclooxygenase 2 (COX-2) is an inducible enzyme that is over-expressed in tumor cells and is known to induce tumor growth in different types of cancers, so delivery of COX-2 inhibitors has a potential role in decreasing tumor progression in SCC [4,5].

Celecoxib (CXB) belongs to cyclooxygenase-selective COX-2 inhibitors with pronounced analgesic and anti-inflammatory activities [6]. Such performance enables CXB to be commonly used for inflammatory bone disorders, e.g., osteoarthritis and rheumatoid arthritis [7,8]. It has also demonstrated an anti-cancer activity for certain kinds of cancer, including skin carcinoma [9], breast tissue cancer [10], colorectal cancer [11], and bladder and lung tumors [12,13]. The oral delivery of CXB showed many concerns regarding its absorption and low bioavailability [14,15,16], in addition to the discovered cardiovascular disorders associated with high doses [14,17,18]. Thus, topical administration could have a beneficial advantage in this regard.

The stratum corneum (SC), the skin’s top layer, is a significant barrier and rate-limiting step for drug diffusion through the skin [19]. Lipid vesicles have shown promise during the last few years for the efficient delivery of different drugs, ranging from liposomes to second-generation vesicles such as transferosomes, ethosomes, and transethosomes [20,21,22]. The second-generation lipid vesicles showed pronounced skin penetration performance due to their constituents compared with conventional liposomes [23,24]. Transferosomes are essentially made of phospholipids and edge activators (EAs) which confer their elasticity, resulting in better skin penetration. Ethosomes are formulated using phospholipids and ethanol. This combination disrupts the stratum corneum (SC), leading to deeper penetration into the skin layers [19]. Transethosomes combine the advantages of both transferosomes and ethosomes due to the presence of both ethanol and an EA. This combination allows for a synergistic mechanism resulting in better penetration through the skin’s SC [25,26].

The objective of this research is to develop CXB -loaded TES within a topical hydrogel system to improve the drug’s skin permeability, allowing for enhanced delivery of CXB to tumor microenvironment to impede cell growth in the tumor cell, as well as minimizing the non-specific distribution in normal cells associated with chemotherapeutics and avoiding the side effect associated with oral administration of CXB. *In vitro* performance of the CXB-TES gel was examined using a skin cancer cell line (A431). Additionally, the optimized preparation was assessed for its safety, DNA damage and fragmentation, and the expression alteration of the cyclin-dependent kinase inhibitor 2A (CDKN2A) gene related to skin cancer progression.

## 2. Materials and Methods

### 2.1. Materials

Celecoxib was kindly obtained from Sedico Pharmaceuticals Co. (October, Cairo, Egypt). Span 60, Tween 80, sodium deoxycholate, hydroxypropyl methylcellulose (HPMC), dialysis membrane, (Spectra/Por^®^, 12,000–14,000 Da), Ethylenediaminetetraacetic acid (EDTA), sodium dodecyl sulphate (SDS), dimethyl sulfoxide (DMSO), MTT Salt: 3-(4,5-dimethylthiazol-2-yl)-2,5-diphenyl tetrazolium bromide and trypsin were obtained from Sigma-Aldrich Co. (St. Louis, MI, USA). Carbopol^®^ 934 (CP) was obtained from BDH Chemicals Ltd. (Bristol, UK). A human skin carcinoma (A431), and a normal human skin fibroblast cell (BJ1) were supplied from American Type Culture Collection (ATCC) (Manassas, VA, USA). Cell supplements, such as Dulbecco modified Eagle-medium (DMEM) and DMEM F12 media (1% L-glutamine, sodium bicarbonate), Foetal bovine serum (FBS), and antibiotic-antimycotic (10,000 U/mL Penicillin-Potassium, 10,000 µg/mL streptomycin sulfate, and 25 µg/mL amphotericin-B) were obtained from Lonza Walkersville, Inc. (Walkersville, MD, USA). All compounds were analytical grade and utilized without additional purification.

### 2.2. Formulation of CXB-Loaded Ethosomes (ES) and Transethsomes (TES)

CXB-entrapped ES and TES were formulated using the previously described cold method [27,28]. Briefly, to prepare CXB-loaded ES, 1% *w/v* CXB along with 300 mg phospholipone 90G were dissolved in 30% *v/v* ethanol under stirring (700 rpm) at room temperature (25 ± 0.1 °C) for 10 min utilizing a magnetic stirrer, followed by addition of deionized water (Qs to 100%) dropwise for a further 5 min. Similarly, TES were prepared using different edge activators, either sodium deoxycholate (SDC), Tween 80 (T80) or span 60 (S60), which were added along with the drug and lipid components. The constituents of the various formulations are summarized in Table 1. Afterward, the obtained lipid vesicles were kept in tightly sealed amber tubes at 4.0 ± 0.5 °C for further studies. Finally, blank TES (CXB-free) was fabricated using the same protocol as a control.

### 2.3. Characterization of CXB-ES and CXB-TES

#### 2.3.1. Vesicle Size and Surface Charge Measurements

A dynamic laser light scattering (DLS) technique was applied to estimate the particle size distribution of the fabricated lipid vesicles at 25.5 ± 0.5 °C, using the Malvern Zeta-sizer Nano-ZS (Malvern Instruments, Worcestershire, UK). Before each measurement, the lipid vesicle suspension dispersed in deionized water at 1 mg/mL and was vortexed for at least one minute. The surface charge of the developed CXB-lipid vesicles was determined using electrophoretic mobility data acquired in deionized water. Measurements were estimated in triplicates.

#### 2.3.2. Entrapment efficiency

The entrapment efficiency (EE%) of CXB within nano ES and TES was determined indirectly by measuring free un-entrapped CXB spectrophotometrically using a Shimadzu UV-1601 PC (Kyoto, Japan), with λ_max_ of 255 nm, after cooling centrifugation, of CXB-nano suspension at 18,000 rpm and 4.0 °C for 30 min, utilizing a large-capacity bench-top refrigerated centrifuge (Sigma Labor Zentrifugen GmbH, Osterode am Harz, Germany). The average value of EE for triplicate samples was calculated using Equation (1):

(1) $$\mathrm{EE}\%\;=\frac{WT-WF}{WT}\times 100$$

where *WT* = weight of the total CXB in the supernatant and sediment, and *WF* = weight of the free un-incorporated CXB quantified in the supernatant, respectively [29].

#### 2.3.3. Evaluation of CXB-Lipid Interaction Using the Differential Scanning Calorimetry (DSC)

To evaluate CXB-lipid interactions, the DSC-thermograms were acquired using a Themys One+ differential scanning calorimeter (Setaram, KEP TECHNOLOGIES, France). Samplings measuring 1–2 mg of CXB powder, Phospholipone-90G (PL-90G), sodium deoxycholate (SDC), blank, dried TES powder, and optimized CXB-TES dried powder (F4) were placed in aluminum pans and heated at a scanning rate of (10 °C/min, 30 to 250 °C) in the presence of nitrogen, with the flow rate set to 40 mL/min. By using CALISTO-thermal analysis software 2.0 (Setaram, KEP TECHNOLOGIES, France), the melting point and enthalpy (DH, Joule/g) can be estimated.

#### 2.3.4. Fourier Transform-Infra-Red Spectroscopy (FTIR)

The chemical attributes of CXB powder, Phospholipone-90G (PL-90G), sodium deoxycholate (SDC), blank, dried TES powder, and optimized CXB-TES dried powder were evaluated using FT-IR spectrophotometry (Alpha II, model: Bruker, USA). Samples (4–5 mg) were mixed with potassium bromide and were compressed into discs. The FT-IR spectrum was recorded from 4000 to 400 cm^−1^.

#### 2.3.5. Morphology

Optimized CXB-TES were imaged employing a transmission electron microscope (TEM) (JEOL 100 CX, Tokyo, Japan) at 80 kV accelerating voltage. The sample was treated by adding 20 µL of the TES suspension onto a Formvar-coated 300 mesh grid stabilized with evaporated carbon film for 1 min. A filter paper was used to remove the excess sample. The aqueous solution of uranyl acetate (2% *w/v*) was used to negatively stain the vesicles by adding 20 µL for a few seconds, followed by drying overnight at room temperature. Images were captured using the AMT-700 camera (Advanced Microscopy Techniques, Woburn, MA, USA).

### 2.4. Preparation of CXB-Loaded Transethosomal (TES) Hydrogel

A CXB-loaded TES formulation (Formula F4) was dispersed in various hydrogel bases, namely hydroxypropyl methylcellulose (HPMC) 2.5% *w/w*, and carpobol-934 (CP) 0.5% *w/w*. To form hydrogels, the polymer was dissolved in deionized water. The aqueous polymer dispersion was hydrated for 5 h until a transparent gel formed. Gel viscosity was determined using a Brookfield DV+ II model LV viscometer at 1.5 rpm and 25 ± 0.1 °C. CXB-loaded TES were centrifuged at 36.670*× g*, 4.0 ± 0.5 °C for 30 min. The acquired pellets were blended with particular amounts of the gels so that the ultimate CXB concentration in the hydrogel was set to 1.0% *w*/*w*. The obtained gels were vortexed until homogeneous [28].

### 2.5. Evaluation of CXB-Loaded Transethosomal Hydrogel

The clarity of the formed gels was examined visually, via inspection of any signs of turbidity or phase separation [30]. In addition, the consistency of the gels was evaluated by pressing the gel between the index finger and thumb. The pH of CXB-TES-loaded hydrogel preparations was assessed via a digital pH meter (Mettler Toledo, Greifensee, Switzerland). The viscosity of CXB-TES hydrogel formulations was estimated using a Brookfield digital DVIII Model viscometer (Brookfield Engineering Laboratories, Inc., Stoughton, MA, USA) at room temperature, 15 rpm, using spindle S-94. All the measurements were carried out in triplicates.

#### Entrapment Efficiency (EE%)

A measure of 0.5 g of CXB-loaded TES gel formulation was dissolved in ethanol to dissolve vesicles and extract the CXB. Absorbance was measured spectrophotometrically at λ_max_ of 255 nm using a spectrophotometer (Shimadzu, Kyoto, Japan). EE% was assessed using the linear regression equation attained from ethanol’s CXB standard calibration curve. The mean percent of EE% was presented as an average of 3 readings.

### 2.6. In Vitro Drug Release Study

*In vitro* drug release from the optimized CXB-loaded TES formulation (Formula F4) compared to CXB-TES-loaded HPMC-based hydrogel, and CXB-TES-loaded-Carbopol-934 based hydrogel was studied as described previously [21,31]. A previously soaked dialysis membrane (Spectra/Por^®^, MWT cutoff 12,000–14,000) was extended over the open bottom end of a glass tube and tightened by a rubber band. A measure of 1 mL of TES suspension (Formula F4) containing 5 mg CXB, or TES gel (1 g equivalent to 5 mg CXB), was placed over the cellulose membrane. The glass tube was submerged in phosphate buffer (pH 6.8, 50 mL) with 1% *w/v* SLS at 37 ± 0.5 °C and shaken at 75 rpm employing a thermostatically controlled water bath shaker (WiseBath, WSP-45, Korea). Aliquots (5 mL) were replaced with release media. The CXB concentration was estimated spectrophotometrically utilizing a Shimadzu UV-1601 PC (Kyoto, Japan), at λ_max_ of 255 nm, at predetermined time points (0.5, 1, 2, 3, 4, 6, 12, and 24 h). *In vitro* release assay was performed in triplicate. Various mathematical models (zero-order, first-order, Higuchi, and Korsmeyer-Peppas) were employed to specify the kinetics and mechanism of CXB release from the developed formulations [32,33,34,35,36].

### 2.7. Ex Vivo Permeation Study

The permeability studies were conducted using rat abdominal skin as per earlier published methods [27,37]. The abdomen skin was cut from 6–8-week Wistar male albino rats weighing 90–100 g. All animals were maintained according to the Subcommittee of the Faculty of Medicine, Assiut University (IRB Local Approval No: 17300867, Date 27 October 2022) and Health Research Ethics recommendations, Qassim University (Approval No: 21-10-07), according to the National Research Council (US) Guide for the Care and Use of Laboratory Animals [38]. The rats’ abdomens were shaved using electric clippers. Then, connective tissues, fats, and subcutaneous tissues were taken off. Skin samples having abnormalities, such as small pores or fissures, were excluded. The skin was rinsed with saline and dried using filter sheets. The skin piece was fitted in the bottom of a test tube to be used as a permeation membrane. The stratum corneum was set to face the donor compartment of the cell, while the dermal side was allowed to be in contact with the receptor compartment of the cell. The donor cell was filled with either CXB-TES-loaded HPMC hydrogel or free CXB-loaded HPMC hydrogel. The method depicted for the *in vitro* drug release test was used. At predefined time intervals (up to 6 h), 3 mL of the release medium was replaced with new media. Analysis of samples was done at λ_max_ of 255 nm. All experiments were done in triplicates. 

The cumulative amount of CXB permeated per unit of skin membrane area (Q_n_, mg/cm^2^) was plotted versus time (h) to construct the permeation profiles [39]. Then, the apparent permeability coefficient (*P_app_* value) was calculated in accordance with the following equation:

(2) $$\mathrm{Papp}\;=\frac{\Delta Q}{\Delta t}\times \frac{1}{\left(C_{o}\times A\right)}$$

where Δ*Q*/Δ*t* is the linear mass appearance rate of the drug of interest in the acceptor chamber, $C_{o}$ is the initial drug concentration in the donor chamber, and *A* is the skin’s surface area (i.e., 0.785 cm^2^). The steady-state flux (*J_ss_*, mg/cm^2^ h) was estimated according to the below equation:

(3) $$J_{ss}=\frac{Amounts\;of\;the\;drug\;permeated}{Time\;\times \;Area\;of\;the\;membrane}$$

### 2.8. In Vitro Proliferation Study

A cytotoxicity test was performed based on the mitochondrial-dependent reduction of yellow MTT (3-(4,5-dimethylthiazol-2-yl)-2,5-diphenyltetrazolium bromide) to insoluble formazan crystals [40]. The assay was applied to examine the cytotoxic influences of five preparations, namely CXB-TES solution (1%), blank HPMC gel, CXB gel (1%), CXB-TES-loaded gel, and CXB powder, on A431 Skin cancer cell lines and BJ1 healthy skin cell lines. First, cells were plated in a fresh growth media at 10 × 10^3^ cells/well for A431 skin cancer cell lines and at 10 × 10^4^ for BJ1 cell lines. Media aspiration was done after 24 h, followed by adding a medium (without serum). Next, the tested preparations or doxorubicin (positive control) were added to cells, followed by incubation for 48 h. Afterward, the medium was substituted with 40 µL MTT salt (2.5 μg/mL), and cells were incubated for another 4 h. Next, a 200 μL of 10% sodium dodecyl sulfate (SDS) in deionized water was put in each well, and then cells were incubated overnight at 37 ± 0.5 °C to pause the reaction and solubilize the resulting crystals. The absorbance was recorded at λ_max_ of 540 nm. Probit analysis was done for IC50 and IC90 calculation using SPSS 11 program.

### 2.9. Gene Expression Analysis by Quantitative RT-PCR

#### 2.9.1. RNA Isolation and Reverse Transcription (RT) Reaction

The total RNA genome was isolated from normal and cancer skin cell cultures using RNeasy Mini Kit (Qiagen, Hilden, Germany) supplemented with DNaseI (Qiagen). The procedures were performed following the company’s protocol. To degrade DNA residues in the isolated total RNA, one unit of RQ1 RNAse-free DNAse (Invitrogen, Germany) resuspended in DEPC-treated water was used. Analysis was done at λ_max_ of 260 nm. The purity of the total RNA was assessed by the absorbance ratio (260/280 nm), which is between 1.8 and 2.1, indicating a pure RNA sample [41]. In addition, the proportion of the ribosomal bands (28S:18S) was used to indicate RNA integrity [42]. Aliquots utilized for the reverse transcription (RT) experiment either immediately or frozen at −80 ± 0.5 °C. Complete Poly(A)^+^ RNA separated from either normal or cancer skin cell lines was reverse transcribed into cDNA, employing a RevertAid TM First Strand cDNA Synthesis Kit (Fermentas, Germany), 20 µL. A master mix containing 5 μg of total RNA was used [43]. The master mix includes 50 mM MgCl_2_, 10× RT buffer (50 mM KCl, 10 mM Tris-HCl, pH 8.3), 10 mM each of dNTP, 50 µM oligo-dT primer, 20 IU ribonuclease inhibitor (50 kDa recombinant enzyme to inhibit RNase activity) and 50 IU MuLV reverse transcriptase. Centrifugation of each mixture was done at 1000× *g* for 30 sec. Afterward, each mixture was transferred to the thermocycler at 25 ± 0.5 °C for 10 min, followed by 1 h at 42 ± 0.5 °C. Finally, a denaturation step at 99 ± 0.5 °C for 5 min was performed. The reaction tubes containing RT preparations were ice-cooled until utilized for cDNA amplification.

#### 2.9.2. Quantitative Real-Time Polymerase Chain Reaction (qRT-PCR)

The qRT-PCR was performed using normal and cancer skin cell lines to get cDNA copy numbers. The StepOne™ Real-Time PCR System was used from Applied Biosystems (Thermo Fisher Scientific, Waltham, MA, USA). The reaction mixture (25 mL) comprised of 1× SYBR^®^ Premix Ex TaqTM (12.5 mL), 0.2 mM sense primer (0.5 mL), 0.2 mM antisense primer (0.5 mL), distilled water (6.5 mL), plus 5 mL of cDNA template [44]. In three-step RTPCR, step one was set to 95.0 °C for 3 min. The next step consisted of 40 cycles, each cycle divided into three steps: (a) at 95.0 °C for 15 s, (b) at 55.0 °C for 30 s, and (c) at 72.0 °C for 30 s. Finally, 71 cycles ranging in temperatures from 60.0 °C (the starting point) to 95.0 °C (the ending point). Following each round of qRT-PCR, melting curve analysis was carried out at 95.0 °C to assure the quality of primers [45]. Each round involved distilled water as a control. The sequences of specific primers of the cyclin-dependent kinase inhibitor 2A (CDKN2A) gene are displayed in Table 2. The ratio of the target’s quantitative characteristics to the reference was calculated using the 2^−ΔΔCT^ method [46,47].

### 2.10. DNA Damage in Normal and Cancer Skin Cell Lines Using the Comet Assay

The comet assay was performed using healthy and tumor skin cell lines. The test was conducted as described previously [48]. Briefly, after the cells were exposed to the tested formulations, the cells were collected and trypsinized. The centrifuged cells (1.5 × 10^4^ cells) were suspended in 0.75% low-gelling-temperature agarose. The cells suspended in agarose were pipetted onto dry pre-coated microscope slides. Then, the slides were immersed in lysing solution at 50 ± 0.5 °C (0.5% SDS, 30 mM EDTA, pH 8.0) for at least 4 h, followed by washing overnight using Tris/borate/EDTA buffer, pH 8.0, at room temperature. Afterward, samples were transferred for electrophoresis at 0.6 V/cm for 25 min, then stained with propidium iodide. The slides were analyzed using a fluorescence microscope equipped with a CCD camera. For slide scoring, Comet images (150 separate images) were analyzed for the percentage of DNA in the comet tail, the tail moment, and the content of DNA. The percent of DNA in the comet tail (100 tested cells/sample) was used to indicate the quantity of DNA damage. A visual score was assigned to each cell based on comet tail length migration and the relative proportion of DNA in the nucleus: class 0 (undamaged DNA, no tail), class 1 (very little DNA damage, comet tail with a length less than the diameter of the nucleus), class 2 (moderate DNA damage, tail lengthened between 1× and 2× the nuclear diameter), and class 3 (highly damaged DNA, tail longer than 2× the diameter of the nucleus) [49].

### 2.11. DNA Fragmentation Assay

The DNA fragmentation test was conducted in normal and cancer skin cell lines accordingly, with the procedure specified by Yawata [50] with some modifications. In brief, healthy, cancerous skin cell lines (approximately 1 × 10^6^ cells) were seeded in different Petri dishes (60 × 15 mm, Greiner) and then exposed to the tested formulations for 24 h. Afterward, all cells (including floating cells) were trypsinized, washed, and lysed. The lysis buffer used to dissolve the cell membranes comprised 10 mM Tris, pH 7.4, 150 mM NaCl, 5 mM ethylenediaminetetraacetic acid (EDTA), and 0.5% Triton X-100 for 30 min on ice. Liquidates were cleaned using a vortex mixer and centrifuged at 10,000× *g* for 20 min. The extraction of fragmented DNA in the supernatant was done using an equal volume of neutral phenol: chloroform: isoamyl alcohol mixture (25:24:1). Quantitative analysis of DNA content was done electrophoretically on 2% agarose gels containing 0.1 μg/mL ethidium bromide.

### 2.12. Statistical Analysis

At least three duplicates of each experiment were conducted, and means±SD were recorded. One-way ANOVA with Tukey Kramer multiple estimates or a two-sided Student’s *t*-test was used to examine the groups’ statistically significant differences (GraphPad Prism 6.0, San Diego, CA, USA).

## 3. Results and Discussion

### 3.1. Characterization of CXB-ES and CXB-TES

Particle size (mean diameter), polydispersity index (PDI), and zeta potential of the developed lipid vesicles are displayed in Table 3. The average particle size of the prepared ES was 363.2 ± 21.6 nm with a high PDI of 0.9 ± 0.03. The incorporation of EA significantly decreases the vesicular size of the prepared TES. The type and concentration of penetration enhancer or edge activator affect TES size [51]. The influence of EA type on particle size may be related to hydrophilic-lipophilic balance (HLB), molecular structure, and ionic nature of the employed surfactant [52]. The HLB values for S60, T80, and SDC were 4.7, 15, and 23.4, respectively [53,54,55]. As the HLB value decreases, the lipophilicity of the EA increases, and the particle size increases [53]. TES prepared using S60 showed larger particle sizes than particles prepared using T80 and SDC. The effect of different concentrations of EA on the particle size is shown in Table 3. As the amount of EA increases from 10 to 30 mg, the vesicular size decreases. The solubilizing properties of these EA thus inhibit vesicle fusion, leading to reduced vesicle size [51]. Increasing the amount of SDC from 10 to 30 mg increases the size of the prepared TES from 53.3 ± 14.2 to 75.9 ± 0.4 nm but decreases the PDI from 0.7 ± 0.05 to 0.4 ± 0.01, indicating more uniformity of the particles [56]. This may be because of the EA’s anionic nature, which causes a significant repulsive force between the vesicle lamellae due to negative charge accumulation on the vesicles, increasing the interior aqueous core [57]. Generally, formulations with a PDI value below 0.5 are considered acceptable.

The zeta potential is a crucial physical characteristic for exploring the stability of vesicles. At the same time, particle aggregation decreases as zeta potential increases more than 30 mV due to the electrostatic repulsion of the charged particles [58]. All preps had negative surface charges. Due to SDC’s negative charge, formulations including it exhibited the greatest zeta potential [19]. Furthermore, the presence of ethanol gives the nanovesicles’ surfaces a negative charge, which improves their colloidal stability [59]. The negative charge of phosphate groups in phospholipone also contributed to the vesicles’ negative zeta potential [60].

### 3.2. Entrapment Efficiency (EE%)

EE% of the prepared TES varied from 77.9 ± 1.5% to 89.4 ± 1.9%. The EE% of formulations containing S60 was higher than the EE% of the formulation prepared using T80. This result could be ascribed to the difference in the used EA’s hydrophilic-lipophilic balance (HLB) values. The higher the lipophilicity of EA (low HLB values), the better the entrapment of lipophilic drugs. CXB is highly lipophilic with a log *p*-value of 3.99, hence S60 increases EE%. Additionally, the interaction between the highly hydrophobic alkyl domains of S60 and the hydrophobic parts of the vesicles prevents drug escape due to condensed layers of vesicles [61].

EA’s lipid phase transition temperature (Tc) impacts EE%; the higher the Tc, the greater its capacity to build a more ordered structure and a less leaky bilayer, which may also increase EE%. Using EAs with a lower Tc may cause abnormal structure formation and increase vesicle bilayer fluidity, limiting drug EE%. This effect appears with a formulation prepared using S60 (F8–F10), which has a high Tc value of 53 °C, showing higher EE% [62]. As the amount of EA increases from 10 to 30 mg, the EE% decreases. This could be attributed to pore formation due to increased surfactant incorporation in the formulation leading to a leaky bilayer, which results in drug escape and eventually decreased EE% [63]. Formulations prepared using SDC deviate from these explanations and showed high EE% and EE% increase with rising concentration. This observation could be due to the electrostatic repelling force, which causes a high inter-bilayer distance leading to increased EE% and improved ethosomal system stability, possibly owing to vesicle surface charge, and thus avoided vesicles agglomeration. This finding is in agreement with the previous study [19]. Therefore, TES formulation (F4) prepared using 30 mg SDC showed the highest stability (zeta potential value of −44.7 ± 1.52), high drug loading (EE% value of 88.8 ± 7.2), good uniformity (PDI value of 0.4 ± 0.01) and small size (P.S. value of 75.9 ± 11.4 nm). Therefore, this formulation was chosen for further studies.

### 3.3. DSC

**Figure 1:**
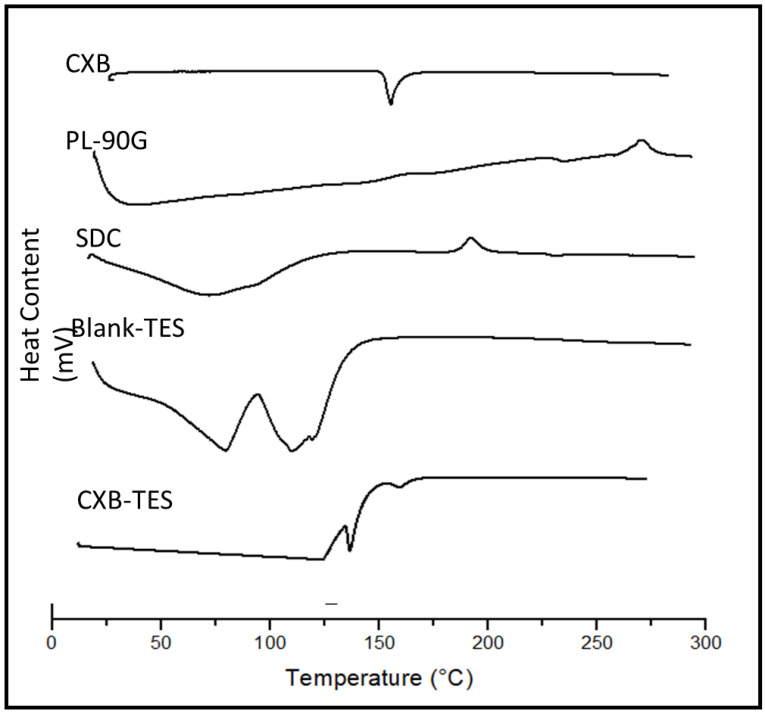
Differential scanning colorimetry (DSC) of CXB powder, PL-90G, SDC, Blank-TES, and the selected CXB-TES (F4).

Figure 1 displays the thermograms of pure CXB powder, PL-90G, SDC, blank-dried TES, and the selected CXB-TES. The DSC scan shows CXB endothermic peak at 163.3 °C approximated to the melting point of pure CXB [64], confirming its crystalline structure. The DSC thermogram of PL-90G displays a phase transition temperature at 41.8 °C [28]. The thermogram of SDC demonstrating a broad endotherm initiated at 110.82 °C might be due to the loss of water molecules, and an exo thermic recrystallization peak existed at 238.0 °C [65]. On the other hand, the selected CXB-TES (F4) shows a decreased intensity of the distinctive peak of the drug. This observation may confirm the entrapment of CXB in lipid vesicles following interactions between the drug and PC and SDC. Such interactions as hydrogen bonding, Van der Waal’s attraction, or dipole-dipole forces might illustrate the formation of homogenous vesicle shape and structure with enhanced physical stability, as previously reported [28,53]. 

### 3.4. FT-IR

**Figure 2:**
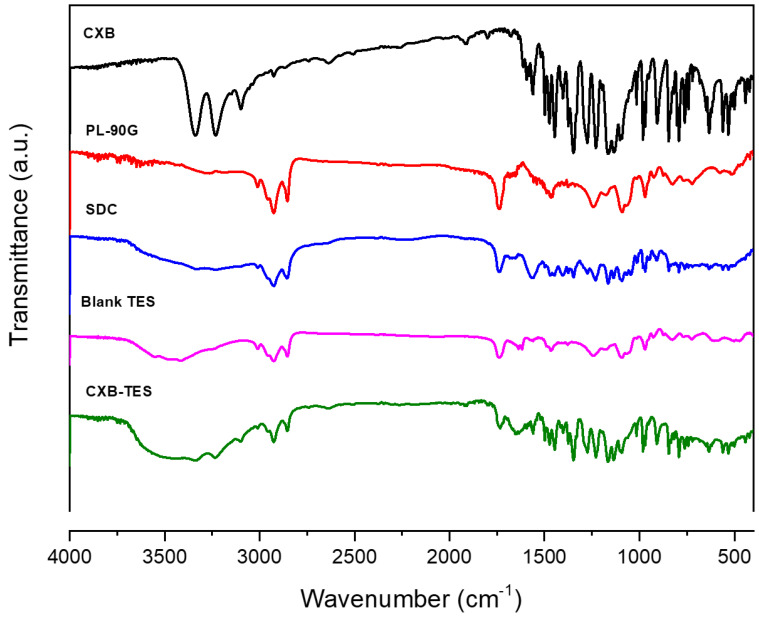
FT-IR spectra of CXB, phospholipone (PL-90G), sodium deoxycholate (SDC), blank TES, and the selected CXB-TES (F4).

For further elucidation and confirmation of the possible interactions and structural changes of CXB with the lipid matrix, the FT-IR spectrum was recorded in the range of 4000–400 cm^−1^ for the drug alone, PL-90G alone, SDC alone, their corresponding physical mixture, and the selected blank and loaded TES (Figure 2). The spectrum of pure CXB showed typical absorption bands at 3160 and 3260 cm^−1^, characteristic of –NH symmetric and asymmetric stretching vibrations, respectively. Distinct bands at 1150 and 1340 cm^−1^ attributed to S=O symmetric and asymmetric stretching, respectively. NH bending vibration was at 1560 cm^−1^_,_ and aromatic –CH bending vibration was at 780 cm^−1^. The findings were consistent with previously reported values [66]. The IR spectrum of PL-90G displays distinctive bands at 2925 and 2854 cm^−1^ for C–H stretching vibrations, a stretching band at 1737 cm^−1^ of the carbonyl group of ester, and a stretching C–O band at 1240 cm^−1^ [67]. Figure 2 displays three specific attributes at 2938.1, 2864.3, and 1561.2 cm^−1^, matching SDC’s CH and COO bands [68].

The FT-IR spectra of the Blank-TES and the CXB-TES preparations were nearly identical. However, CXB incorporation into the lipid matrix might cause the reduced intensity or slight shifting of CXB characteristic bands owing to hydrogen bonds, Van der Wall forces, or dipole interactions, indicating CXB encapsulation into the lipid matrix and nano-vesicle stability [69,70]. In addition, the drug’s dissolution in lipids affects CXB entrapment in the prepared TES [71]. 

### 3.5. Morphological Analysis

**Figure 3:**
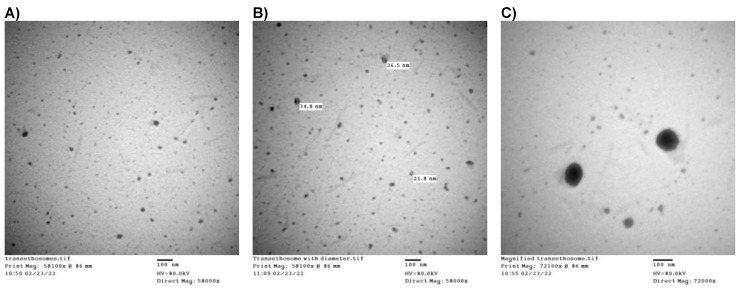
TEM images of the selected CXB-TES formulation (F4). The scale bar represents 100 nm. (**A**) TEM image of the CXB-TES, which showed spherical NPs appearing as spots, (**B**) Size measurement of the CXB-TES by TEM, and (**C**) CXB-TES with high magnifications 72,000×.

The TEM photos of the optimum CXB-TES preparation (F4) are shown in Figure 3. The vesicles exhibit irregular spherical structures with a homogenous size distribution to a high extent. The observed morphology could be attributed to the presence of EA in the composition, which confers to the elasticity of the prepared lipid vesicles [19]. The particle size received from TEM images was smaller than that obtained from DLS measures. This disparity can be attributed to those particles in aqueous suspension showing a mild degree of aggregation, while particles imaged in TEM are homogenous and not aggregated [72]. Additionally, TEM images reflect the size of the particles after the dryness of the surrounding hydrated layer, resulting in smaller size measurements than DLS [73,74,75]. DLS gives the hydrodynamic diameter of suspended hydrated vesicles, usually higher than the size of the dry vesicles captured by TEM [75].

### 3.6. Characterization of CXB-Transethosomal Hydrogel

The prepared TES hydrogels were homogenous, clear with no inspection of phase separation without appreciable lumps, and uniform in consistency. The pH of the prepared TES gel was 6.5, and 5.8 for HPMC-loaded and CP-934 loaded TES hydrogels. Furthermore, high CXB contents were found in the prepared TES hydrogels: 99.5 ± 2.5 and 98.99 ± 3.7% for HPMC and CP-934 TES hydrogels, respectively. This high EE% was acceptable and approved the formulation technique. The viscosity of HPMC 2.5% *w/w* loaded TES hydrogel containing CXB was 6400 ±150 cPs, while the viscosity of carbopol 934 0.5% *w/w* loaded TES hydrogel containing CXB was 15,500 ± 100 cPs.

### 3.7. In Vitro Drug Release

**Figure 4:**
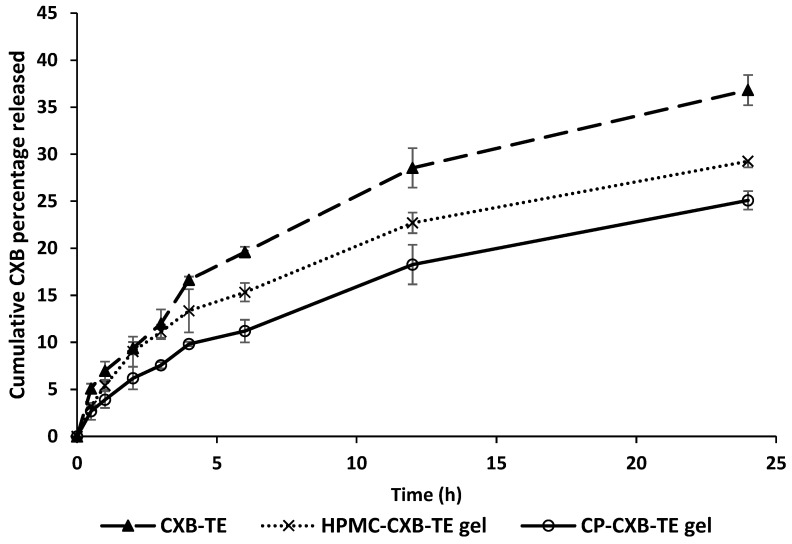
*In vitro* release profile of CXB-transethosomes (CXB-TES) formulation compared to CXB transethosomes-loaded HPMC-based hydrogel (HPMC-CXB-TES gel), and CXB transethosomes-loaded Carbopol-934-based hydrogel (CP-CXB-TES gel), in phosphate buffer (pH 6.8, 1% *w/v* SLS) at 37 ± 0.5° C. Data are expressed as mean ± S.D. (*n* = 3).

The *in vitro* release study of CXB from the optimized TES formulation (F4) was carried out in PBS (pH 6.8, containing 1% *w/v* SLS) at 37 ± 0.5 °C, employing a modified dialysis membrane diffusion technique [20,66]. The *in vitro* release profile of CXB-TES was compared with that of CXB-TES-loaded HPMC-based gel and CXB-TES-loaded Carbopol-934-based gel (Figure 4). Generally, CXB-TES suspension showed a higher *in vitro* release than the hydrogel preparations.

After 24 h, the cumulative release from CXB-TES suspension was 36.81 ± 0.79%, which was significantly higher than that released from HPMC and CP 934-based TES hydrogel (*t*-test; *p* ≤ 0.05). After 24 h, the cumulative drug release followed this order: TES suspension > TES HPMC hydrogel > TES Carbopol-934 hydrogel. The differences were statistically significant (*t*-test; *p* ≤ 0.05). Similar conclusions were observed previously, where the vesicle dispersion within different gel bases led to the subsequent reduction in drug release rates [76,77,78]. Drug diffusion from vesicular carriers laden in gel bases occurs in two stages: drug release from the reservoir vesicles followed by drug diffusion across the gel structure [79]. Gel viscosity affects the drug release rate, with higher gel viscosity reducing the drug release rate [80,81].

To actually investigate the mechanisms promoting CXB release from various preparations, kinetic analysis was done by fitting data to either a zero-order, first-order, Higuchi diffusion, or Korsmeyer–Peppas model [82,83]. The release rate constant (k) and correlation coefficient (R^2^) calculated by various mathematical models are represented in Table S1 (Supplementary Materials Tables S1–S3 and S5).

It was found that the best fit model for CXB release from the developed hydrogels was the Higuchi diffusion model. The estimated *n* values were 0.5 < *n* < 1.0, indicating anomalous non-Fickian drug diffusion. The drug transport mechanism is controlled by gel erosion, and diffusion is merged with the distention of lipid bilayers [33]. These findings agree with previous studies that demonstrated matching release patterns for TES-based hydrogel [84,85]. The constant release of CXB might be attributed to diffusion from the hydrogel network structure and partitioning via TES vesicles.

### 3.8. Ex Vivo Permeation Study

**Figure 5:**
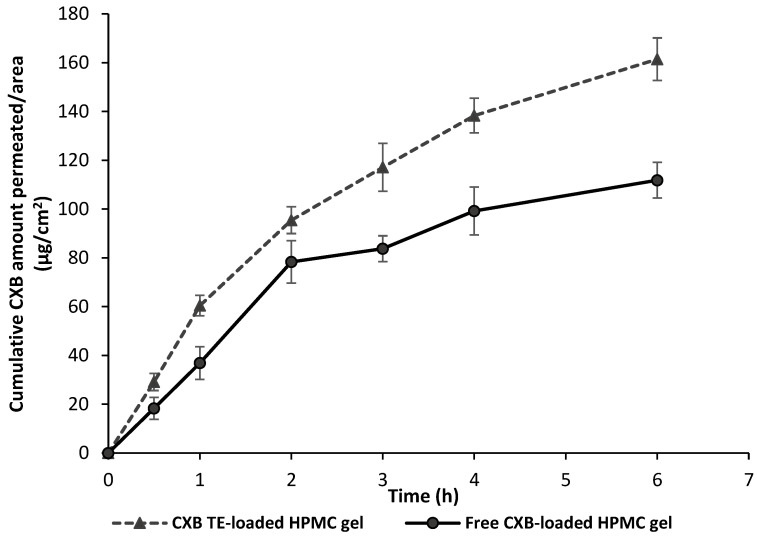
Ex-vivo permeation profile of selected CXB transethosomes-loaded HPMC-based hydrogel (CXB-TES-HPMC gel), in comparison to free CXB-loaded HPMC hydrogel in phosphate buffer (pH 6.8, 1% *w/v* SLS) at 37 °C. Data are expressed as mean ± S.D. (*n* = 3).

Freshly excised rat skin was used as an *in vitro* model for comparing transdermal permeation properties of CXB-TES-loaded HPMC hydrogel and free CXB-loaded HPMC hydrogel to understand the in vivo performance of the optimized TES-based hydrogel preparation (Figure 5 and Table 4). The cumulative amount of CXB permeated as TES gel formulation was significantly higher (161.24 ± 1.17μg/cm^2^ after 6 h) compared to 111.81 ± 3.31 μg/cm^2^ drug permeation from the free CXB-loaded gel formulation at the same time (*t*-test; *p*≤ 0.05). The *in vitro* permeation parameters also revealed that the TES-based gel formulation showed significantly (*t*-test; *p* ≤ 0.05) higher transdermal flux (*Jss*) compared to the free drug-loaded gel formulation, as presented in Table 4. The apparent permeability coefficient (*Papp*) calculated for the transport of drug-containing TES gel across the skin is 2.394 × 10^−2^ ± 0.102 cm/h. The *Papp* value calculated from the linear flux of free drug-containing gel across the skin membrane is 1.702 ± 0.12 × 10^−2^ cm/h, equivalent to a 1.4-fold increased transfer rate of CXB when encapsulated as TES hydrogel.

The characteristics of the gel, accompanied by the size and vesicular nature of TES, are considered the primary reason for this observation. The greater penetration of the sodium deoxycholate TES gel may result from its decreased size, negative zeta potential, and improved fluidity of the lipid bilayers. Various methods, including adsorption and diffusion of vesicles onto the skin’s surface and that the vesicles operate as penetration enhancers that lower the barrier characteristics of the stratum corneum, might demonstrate the capacity of vesicles to facilitate drug transfer through the skin [86]. The vesicular carrier (transethosome) adheres to lipid lamella after interacting with the disturbed layer of the stratum corneum. Due to their elasticity, vesicles may travel across unlimited intercellular passages when ethanol and edge activators are present. [53,87,88]. As established by the experimental results, applying CXB-TES-HPMC hydrogel as a topical delivery system could potentially alleviate future skin cancer development and progression.

### 3.9. In Vitro Proliferation Study

The cytotoxicity of various formulations was tested on the human skin cancer cell line (A431) at 100 µg/mL to prove the activity of CXB-TES against cancer cells. The MTT test was used to compare their findings to those of a normal skin fibroblast cell line (BJ1). The results revealed that CXB-TES suspension, CXB-TES hydrogel, and CXB powder exhibited 100% mortality with IC_50_ 1.4, 17.5, and 1.8, respectively. However, in the case of blank gel and CXB gel, the mortality on A431 cell lines were 10.3% and 18.4% at 100 µg/mL, as depicted in Table 5. The superior efficacy of CXB-TES dispersion may be attributed to the enhanced permeability through the cell membrane. Notably, TES are characterized by a peculiar elasticity attributed to ethanol, which makes vesicles pliable, thus improving their permeation.

Moreover, ethanol might destabilize cell membranes, facilitating their entry [89]. The high cytotoxic activity of CXB (either in free form or encapsulated form) can be ascribed to the anti-inflammatory, antiproliferative, chemo-preventive, and anti-angiogenic activity of CXB [90]. The higher IC_50_ of TES-based gel might be attributed to the inclusion of CXB-TES within the network structure of HPMC gel, leading to a slower flux and permeability with a more sustained release compared to CXB-TES dispersion and free CXB powder that could permeate more freely through the skin [91].

Comparing the effect of TES suspension, TES hydrogel, and CXB powder on normal cell lines (Bj1), the results indicated that CXB powder has the same cytotoxicity on normal and cancer cell lines with relatively similar IC_50_: 1.6 and 1.8 µg/mL, respectively. On the other hand, TES hydrogel has a lower cytotoxic effect on the normal cell with IC_50_ equal to 101.1 µg/mL, and TES suspension has IC_50_ equal to 82.7 µg/mL. This elucidated that the TES gel is safer than CXB powder by 100-fold, indicating an important advantage of the developed formulation for anti-cancer therapeutic. Remarkably, the possibility of applying CXB as TES gel can represent an innovative approach to hamper the toxicity issues related to free drug application.

### 3.10. Gene Expression in Normal and Cancer Skin Cell Lines

**Figure 6:**
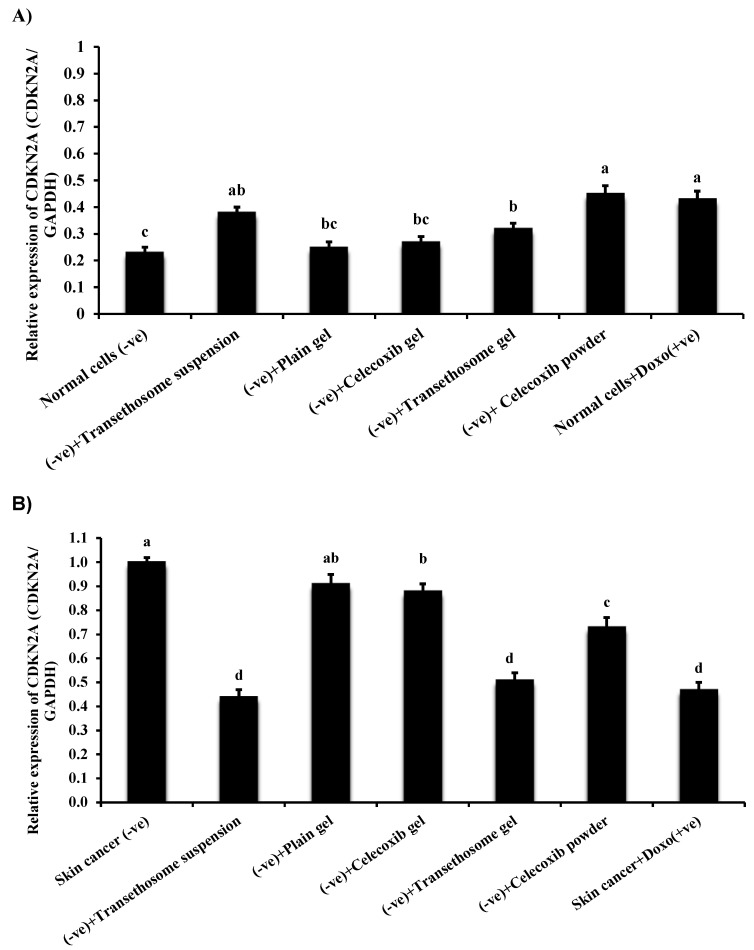
(**A**) The alterations of *CDKN2A* gene in normal skin cell lines treated with different CXB preparations. (**B**) The alterations of *CDKN2A* gene in skin cancer cell line lines treated with different CXB preparations. Data are presented as mean ± SD. ^a,b,c,d^: Mean values within tissue with unlike superscript letters were significantly different (*p* ≤ 0.05; ANOVA/Tukey).

Gene expression analysis was performed on normal and cancerous skin cell lines using skin cancer-related genes, namely the cyclin-dependent kinase inhibitor 2A (CDKN2A) gene (Figure 6a,b). The results revealed that the expression levels of the CDKN2A gene were increased significantly (*p* ≤ 0.05; ANOVA/Tukey) in normal skin cell lines treated with TES suspension and CXB powder, respectively, compared with untreated normal skin cell lines (Figure 6a). Moreover, the expression levels of the CDKN2A gene were elevated considerably (*p* ≤ 0.05; ANOVA/Tukey) in normal skin cell lines treated with doxorubicin compared to negative control normal cell lines. In addition, treatment of normal skin cell lines with TES hydrogel increased the expression levels of the CDKN2A gene compared with negative control normal cell lines. Still, the expression levels of the TES gel-treated group were lower than those in the TES suspension and CXB powder groups.

On the other hand, the expression levels of the CDKN2A gene in groups of normal skin cell lines treated with blank HPMC gel and CXB gel were relatively near to the CDKN2A expression levels in negative control normal cell lines. Expression levels of the CDKN2A gene in skin cancer cell lines are summarized in Figure 6b. The results showed that the expression levels of the CDKN2A gene were decreased significantly (*p* ≤ 0.01; ANOVA/Tukey) in skin cancer cell lines treated with TES suspension, TES gel, and doxorubicin (+ve control), respectively, compared with negative control skin cancer cell lines. Furthermore, the expression levels of the CDKN2A gene declined considerably (*p* ≤ 0.05; ANOVA/Tukey) in skin cancer cell lines treated with CXB powder compared to negative control skin cancer cell lines. Treatment of skin cancer cell lines with CXB gel slightly decreased the expression levels of the CDKN2A gene compared with negative control cancer cell lines. In contrast, the expression levels of the CDKN2A gene in a group of skin cancer cell lines treated with blank HPMC gel were relatively similar to the CDKN2A expression levels in negative control skin cancer cell lines.

Melanoma is a complicated disorder, comprising environmental, phenotypic, and hereditary risk factors [92]. Approximately 5–10% of melanoma cases ran in families [93]. CDKN2A is related to melanoma susceptibility. Germline modifications in the CDKN2A gene have been reported in 20–40% of melanoma-liable families [94]. The present study proved that the expression of CDKN2A in normal skin cells with different forms of CXB were lower than in skin cancer cells. Furthermore, the levels of expression of the CDKN2A gene were reduced significantly with different forms of CXB, especially with CXB gel < CXB powder < TES hydrogel < TES suspension, explaining that TES hydrogel and TES dispersion are good carriers for improving topical delivery of CXB to the cancer cells, suppressing their progression [23].

Switching off the CDKN2A tumor suppressor gene’s expression and encoding the p16INK4a protein is linked with the emergence of different cancers. The p16INK4a protein contributes a vital role in the cell cycle and senescence by regulating the cyclin-dependent kinase (CDK) 4/6 and cyclin D complexes. Genetic and epigenetic deviations of the CDKN2A gene result in increased tumorigenesis and metastasis with a poor prognosis. In this scenario, restoring genetic and epigenetic reactivation of CDKN2A is a practical strategy for the inhibition and cure of cancer. Previously, Chin-Cheng Su et al. [95] investigated the mechanism of curcumin inhibition of the Retinoblastoma (RB) signaling pathway in DBTRG glial cells. Treatment with curcumin was found to upregulate CDKN2A/p16 and downregulate the phosphorylated RB protein. Competition exists between the CDKN2A/p16 protein and cyclin D1 to bind to the CDK4/6 protein, stopping the phosphorylation of the RB protein. The unphosphorylated RB protein could not detach from its repressor E2F to allow transcription of G1 genes for progressing to the S phase.

### 3.11. DNA Damage in Skin Cell Lines

**Figure 7:**
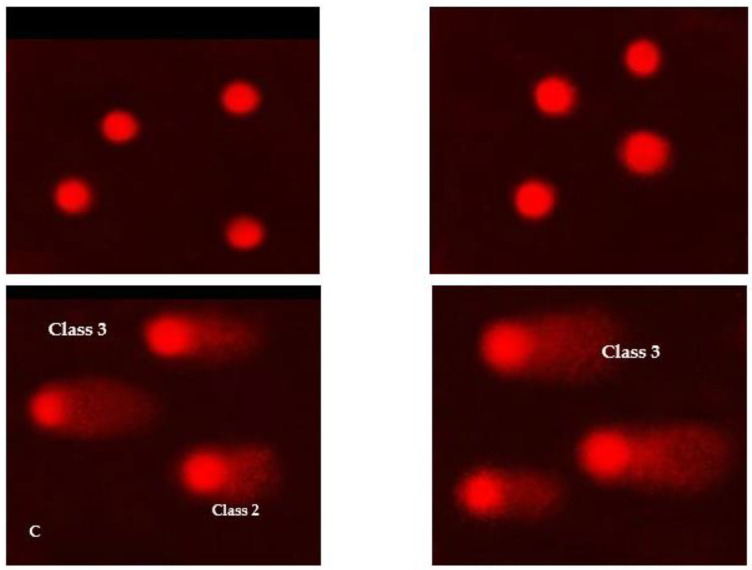
Visual score of normal DNA; (**A**) class 0 and damaged DNA (**B**) class 1, (**C**) class 2 & 3, and (**D**) Class 3 using comet assay in skin cell lines.

The DNA damage in normal and cancer skin cell lines was determined using comet assay, as shown in a Figure 7A–D. The results showed that treatment of normal skin cell lines with CXB powder induced significantly (*p* ≤ 0.01; ANOVA/Tukey) the highest DNA damage values (23.63 ± 0.93%) compared with the control skin cell lines (6.82 ± 0.86%) (Supplementary Material Table S5). In addition, the DNA damage values were increased significantly (*p* ≤ 0.01; ANOVA/Tukey) in normal skin cell lines treated with doxorubicin compared with the control skin cell lines, but these DNA damage values were lower than those in the group treated with CXB powder. Treatment of normal skin cell lines with TES suspension and TES gel exhibited a similar rise (*p* ≤ 0.05; ANOVA/Tukey) in the DNA damage values compared to those in the control group. However, treatment of normal skin cell lines with blank gel and CXB gel showed DNA damage values similar to those in the control group. The damage to the DNA in skin cancer cell lines is summarized in Supplementary Material Table S6. The results showed that treatment of skin cancer cell lines treated with CXB powder induced significantly (*p* ≤ 0.01; ANOVA/Tukey) the highest DNA damage values (27.63 ± 1.21%) compared with negative skin cancer cell lines (11.21 ± 0.87%) (Supplementary Material Table S6). Furthermore, the DNA damage values were increased significantly (*p* ≤ 0.01; ANOVA/Tukey) in skin cancer cell lines treated with doxorubicin (24.82 ± 0.80%) and TES suspension (23.43 ± 0.93%) compared with negative skin cancer cell lines, but these DNA damage values were lower than those in the group treated with CXB powder. Treatment of skin cancer cell lines with TES gel exhibited a considerable rise (*p* < 0.05; ANOVA/Tukey) in the DNA damage values (21.41 ± 0.51%) in comparison to those in the control group (11.21 ± 0.87). However, treatment of skin cancer cell lines with blank gel and CXB gel induced DNA damage values (13.82 ± 0.66% and 16.23 ± 1.07%, respectively) lower than other compound treatments with those in negative cancer cell lines (Supplementary Material Tables S7 and S8).

In the same line with our observations, Liu et al. [96] proved that CXB damages DNA in MCa-35 murine mammary and A549 human lung cancer cells. Induced DNA damage activates p53 signaling (phosphorylation at Ser 15 and Ser 20) and subsequent transcriptional activation of p53 response genes (including p21, GADD45, BAX, PUMA, Bcl2, and NOXA), thus provoking cell cycle arrest and/or apoptosis [97]. Kang et al. [97] demonstrated that CXB enhances glioma cytotoxicity by the induction of DNA damage and p53-dependent G_1_ cell cycle arrest, followed by p53-dependent autophagy.

CXB-loaded liposomes have been shown to effectively induce apoptosis in rat hepatocytes compared to CXP powder [98]. Furthermore, CXB may quickly increase cancer cells’ mitochondrial superoxide production, resulting in widespread ROS-dependent death in murine melanoma B16F10 cells [99]. In addition, COX-2 is necessary for synthesizing prostaglandin E2, which promotes the development of cancerous cells [100,101]. By deactivating myeloid suppressor cells, which promote invasion and angiogenesis, CXB suppresses the development of cutaneous squamous cell carcinoma and basal cell carcinoma [102]. In addition, CXB could suppress the epithelial-mesenchymal transition, a process by which tumor cells weaken intercellular adhesions and enhance their penetration into surrounding tissues [103].

Prior studies indicated that different targets of NSAIDs, besides COX-2, may exist, such as 15-lipoxygenase-1, AKT/PKB Kinase, PPAR delta, and P21 Ras. In addition, calcium ion homeostasis, nuclear factor kB (NFkB), and inducible nitric oxide synthase may play an essential role in apoptosis induction by CXB [104,105,106].

### 3.12. Assessment of the DNA Fragmentation in Normal and Cancer Skin Cell Lines

**Figure 8:**
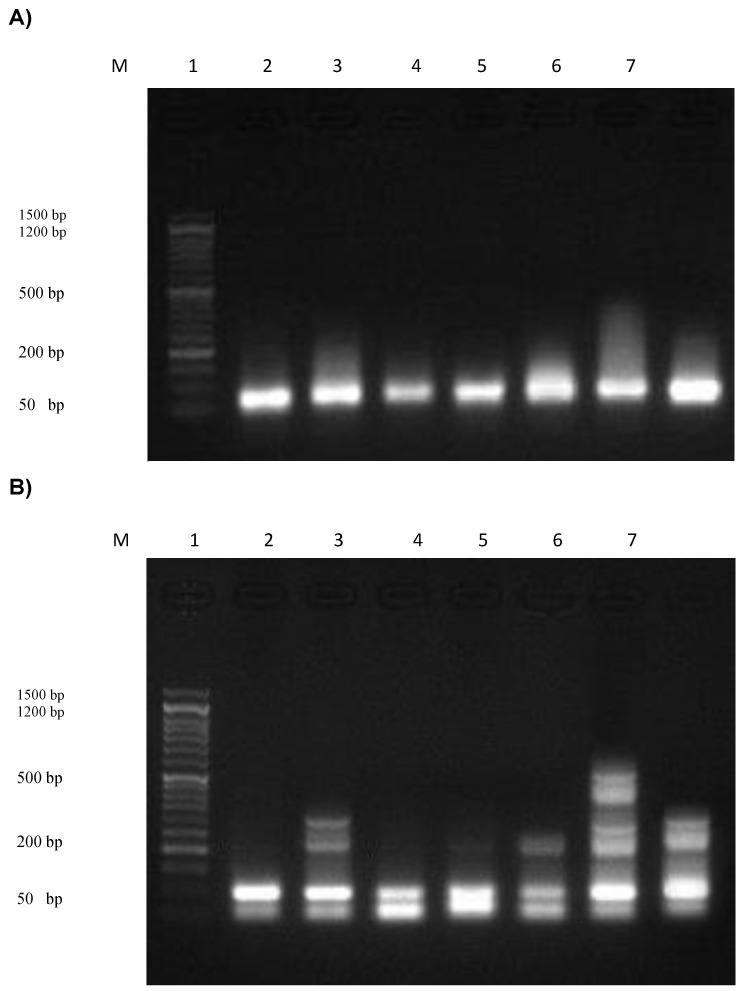
DNA fragmentation was detected with Agarose gel in normal skin cell lines (**A**) and skin cancer cell lines (**B**) treated with different CXB compounds. M: represent DNA marker, Lane 1: represents negative normal skin cell lines (-ve), Lanes 2-6: represent normal skin cell lines treated with TES suspension, blank HPMC hydrogel, CXB hydrogel, CXB-TES hydrogel, and CXB powder, respectively, Lane 7: represents positive skin cell lines treated with doxorubicin (+ve control).

The rates of DNA fragmentation in normal and cancer skin cell lines are summarized in Figure 8a,b, respectively. The results showed that normal skin cell lines treated with CXB powder exhibited a highly significant (*p* ≤ 0.01; ANOVA/Tukey) increase (27.6 ± 0.54%) in DNA fragmentation rates compared with those in untreated normal skin cell lines (8.1 ± 0.24%). Additionally, the DNA fragmentation values were increased significantly in normal skin cell lines treated with doxorubicin (21.7 ± 0.66%), TES suspension (20.6 ± 0.56%), TES hydrogel (19.4 ± 0.48%), CXB hydrogel (17.1 ± 0.23%), and blank HPMC gel (15.2 ± 0.30%) compared with those in untreated normal skin cell lines (8.1 ± 0.24%). Moreover, the agarose gel in Figure 8a exhibited that the DNA fragmentation bands were shown in normal skin cell lines + CXB powder > normal skin cell lines + doxorubicin > normal skin cell lines + TES suspension > normal skin cell lines + TES hydrogel > normal skin cell lines + CXB hydrogel > normal skin cell lines + blank HPMC hydrogel. Figure 8b shows the DNA fragmentation in skin cancer cell lines treated with different CXB preparations. The results showed that the skin cancer cell line treated with CXB powder exhibited the most significant (*p* ≤ 0.01; ANOVA/Tukey) rise (43.7 ± 0.53%) in the DNA fragmentation rates compared with those in untreated skin cancer cell lines (13.7 ± 0.45%). Furthermore, the DNA fragmentation rates were increased significantly in skin cancer cell lines treated with TES suspension (35.5 ± 0.58%), doxorubicin (33.8 ± 0.48%), TES hydrogel (31.6 ± 0.61%), CXB gel (21.2 ± 0.66%), and blank HPMC hydrogel (17.9 ± 0.15%) compared with those in untreated skin cancer cell lines (13.7 ± 0.45%). Additionally, the agarose gel in Figure 8b exhibited that the DNA fragmentation bands were shown in skin cancer cell lines + CXB powder > skin cancer cell lines + TES suspension > skin cancer cell lines + doxorubicin > skin cancer cell lines + TES hydrogel > skin cancer cell lines + CXB gel > skin cancer cell lines + blank HPMC gel (more detail in Supplementary Materials Tables S7 and S8).

Similarly, Ramer et al. [107] indicated that CXB caused DNA fragmentation and lung cancer cell death, suggesting that COX-2 plays a role in its antitumor activity. CXB also increased the cleavage of caspases 9, 8, and 3, which break DNA in cancer cells. Furthermore, treatment of H460/V1 cells with 50 μ M CXB increased the amount of DNA fragmentation compared to untreated cells [108]. Based on the experimental findings, encapsulating CXB into TES containing hydrogel can markedly enhance the anti-tumor effect of CXB in skin cancer cell lines. The anti-tumor potential activity of this anti-inflammatory drug appears to be attributed to its suppression of COX-2 expression, besides the presence of other molecular targets. Consequently, our results show a novel approach for enhancing chemotherapeutic drug efficacy and overcoming the systemic side effects of this drug.

## 4. Conclusions

This study assessed a new therapeutic approach for skin cancer management. TES were prepared by the established cold method with different edge activators. The optimized vesicles were spherical and displayed a high entrapment efficiency (88.8 ± 7.2%), nanometric size, and negative zeta potential. Loading the developed CXB-TES within hydrogel formulation showed more efficiency in controlling drug release compared to TES dispersion. Additionally, the application of TES hydrogel strongly augmented CXB penetration and flux into rat skin approximately 1.4-fold relative to free CXB hydrogel. The developed TES hydrogel significantly reduced A431 cancer cell viability (IC_50_ value of 17.5 μg/mL) while expressing a pronounced safety against normal skin cell lines compared to free CXB. Moreover, the level of CDKN_2_A gene expression was significantly (*p* ≤ 0.01, ANOVA/Tukey) decreased in skin cancer cell lines relative to normal skin cell lines, indicating that TES are a good carrier for topical delivery of CXB to the cancer cells, suppressing their progression. CXB-TES hydrogel induced DNA fragmentation and apoptosis in skin cancer cells. The superior physicochemical performance of the hydrogel, combined with the elasticity and sustaining drug release effect of the TES, could improve skin retention capacity and penetration. Consequently, using TES hydrogel as a potential carrier for the topical delivery of CXB is considered a promising strategy for localized management of skin cancer with the ability to overcome reported adverse effects. The future perspectives will focus on clinical investigations into the therapeutic effect of CXB-TES hydrogel in humans.

## Figures and Tables

**Table 1:** Composition of the prepared CXB-ES and CXB-TES.

Formula Number	Type of Nanovesicles	Type of EA	Amount of EA (mg)
**F1**	Ethosome	NA	NA
**F2**	Transethosome	Na deoxycholate	10
**F3**	Transethosome	Na deoxycholate	20
**F4**	Transethosome	Na deoxycholate	30
**F5**	Transethosome	Tween 80	10
**F6**	Transethosome	Tween 80	20
**F7**	Transethosome	Tween 80	30
**F8**	Transethosome	Span 60	10
**F9**	Transethosome	Span 60	20
**F10**	Transethosome	Span 60	30

EA: Edge activator.

**Table 2:** Primer’s sequence used for *qRT-PCR* of normal and cancer skin cell lines.

Gene	Primer Sequence	GenBank (Accession No.)
**CDKN2A**	F: CAC CCC GCT TTC GTA GTT TT R: CCA ACA CAG TGA AAA GGC AGA	NM_058195.4
**GAPDH**	F: CCA AGG AGT AAG ACC CCT GG R: TGG TTG AGC ACA GGG TAC TT	NM_001256799.3

CDKN2A: Cyclin-dependent kinase inhibitor 2A, GAPDH: glyceraldehyde-3-phosphate dehydrogenase.

**Table 3:** Entrapment efficiency, particle size, PDI, and zeta potential values for the prepared Formulations of CXB-ES and CXB-TES (*n* = 3 ± S.D.).

Formula Number	EE (%)	Particle Size (nm)	PDI	Zeta Potential (mV)
**F1**	59.9 ± 3.4	363.2 ± 21.6	0.9 ± 0.03	−21.5 ± 0.02
**F2**	86.2 ± 2.6	53.3 ± 14.2	0.7 ± 0.05	−34.3 ± 0.58
**F3**	87.9 ± 4.0	70.2 ± 8.8	0.7 ± 0.08	−43.3 ± 0.57
**F4**	88.8 ± 7.2	75.9 ± 11.4	0.4 ± 0.01	−44.7 ± 1.52
**F5**	80.4 ± 2.5	105.0 ± 2.3	0.3 ± 0.08	−22.0 ± 1.00
**F6**	78.5 ± 1.4	92.2 ± 2.3	0.9 ± 0.01	−24.0 ± 0.01
**F7**	77.9 ±1.5	85.0 ± 3.4	0.7 ± 0.11	−18.6 ± 1.52
**F8**	89.4 ± 1.9	143.6 ± 11.8	0.5 ± 0.03	−20.7 ± 0.58
**F9**	87.1 ± 2.0	138.5 ± 2.1	0.7 ± 0.07	−19.3 ± 0.58
**F10**	86.1 ± 4.5	95.7 ± 5.9	0.6 ± 0.04	−23.3 ± 1.52

**Table 4:** Permeability parameters obtained from ex-vivo permeation studies of CXB TES-loaded HPMC hydrogel versus free CXB-loaded HPMC hydrogel across rat skin.

Formulation	Papp × 10^−2^ (cm/h)	Jss (µg/cm^2^/h)	Drug Permeated after 6 h (µg/cm^2^)	ER ^a^
**CXB TES-loaded HPMC gel**	2.394 ± 0.102 *	26.33 ± 1.9 *	161.42 ± 1.17 *	1.4
**Free CXB-loaded HPMC gel**	1.702 ± 0.2	18.72 ± 1.3	111.81 ± 3.31	----

Each value represents mean ± SD (*n* = 3). Abbreviations: Jss, steady state flux; ER, enhancement ratio. * Significant difference from free drug-loaded gel preparation (*t*-test; *p* ≤ 0.05). ^a^ Enhancement ratio (ER) was calculated as Papp (TES gel formulation)/Papp (free drug-loaded gel preparation).

**Table 5:** Cytotoxicity effect of the different tested formulations at 100µg/mL on A431 and Bj1 cell lines and IC_50_ for active compounds.

Formulations	% Mortality at 100 µg/mL (A431 Cell Line)	IC_50_ (µg/mL)	% Mortality at 100 µg/mL (Bj1 Cell Line)	IC_50_ (µg/mL)
**CXB-TES dispersion**	100	1.4	54.3	82.7
**Blank HPMC gel**	10.3	------	22.5	-----
**Free CXB gel 1%**	18.4	-----	31.8	------
**CXB -TES HPMC gel 1%**	100	17.5	43.9	101.1
**CXB powder**	100	1.8	100	1.6
**Negative control**	1	----	2.3	-----
**Positive control (doxorubicin)**	100	23.5	100	18.9

## Data Availability

All data are included in the manuscript.

## Supplementary Materials

The following supporting information can be downloaded at: https://www.mdpi.com/article/10.3390/pharmaceutics15010022/s1, Table S1: Kinetic analysis for release of CXB-TES- carbopol 934 hydrogel; Table S2: Kinetic analysis for release of CXB-TES-HPMC hydrogel; Table S3: Korsmeyer-Peppas Kinetic analysis for release of CXB-TES-HPMC hydrogel; Table S4: Korsmeyer-Peppas Kinetic analysis for release of CXB-TES- carbapol hydrogel; Table S5: Visual score of DNA damage in normal skin cell lines treated with different CXB preparations; Table S6: Visual score of DNA damage in skin cancer cell lines treated with different CXB preparations; Table S7: DNA fragmentation detected in normal skin cell lines; Table S8: DNA fragmentation detected in cancer skin cell lines.

## References

[1] Simões M.F., Sousa J.S., Pais A.C. (2015). Skin cancer and new treatment perspectives: A review. Cancer Lett..

[2] Gan B.K., Yong C.Y., Ho K.L., Omar A.R., Alitheen N.B., Tan W.S. (2018). Targeted delivery of cell penetrating peptide virus-like nanoparticles to skin cancer cells. Sci. Rep..

[3] Hu J.K., Suh H.-W., Qureshi M., Lewis J.M., Yaqoob S., Moscato Z.M., Griff S., Lee A.K., Yin E.S., Saltzman W.M. (2021). Nonsurgical treatment of skin cancer with local delivery of bioadhesive nanoparticles. Proc. Natl. Acad. Sci. USA.

[4] Tołoczko-Iwaniuk N., Dziemiańczyk-Pakieła D., Nowaszewska B.K., Celińska-Janowicz K., Miltyk W. (2019). Celecoxib in cancer therapy and prevention–review. Curr. Drug Targets.

[5] Gao L., Wang T.H., Chen C.P., Xiang J.J., Zhao X.-B., Gui R.-Y., Liao X.-H. (2021). Targeting COX-2 potently inhibits proliferation of cancer cells in vivo but not in vitro in cutaneous squamous cell carcinoma. Transl. Cancer Res..

[6] Fouad E.A., Yassin A.E.B., Alajami H.N. (2015). Characterization of celecoxib-loaded solid lipid nanoparticles formulated with tristearin and softisan 100. Trop. J. Pharm. Res..

[7] Davies N.M., McLachlan A.J., Day R.O., Williams K.M. (2000). Clinical pharmacokinetics and pharmacodynamics of celecoxib. Clin. Pharmacokinet..

[8] Tindall E. (1999). Celecoxib for the treatment of pain and inflammation: The preclinical and clinical results. J. Osteopath. Med..

[9] Sohrabi M., Soleimani J., Roshangar L., Vatansever S., Arbabi F., Khaki A.A., Abbasi M.M., Dustar Y., Javadzadeh Y. (2009). The effect of dietary and topical celecoxib on 4-nitroquinoline-1-oxide-induced lingual epithelium alternations in rat. JPMA.

[10] Mojeiko G., de Brito M., Salata G.C., Lopes L.B. (2019). Combination of microneedles and microemulsions to increase celecoxib topical delivery for potential application in chemoprevention of breast cancer. Int. J. Pharm..

[11] Han Y., Chen P., Zhang Y., Lu W., Ding W., Luo Y., Wen S., Xu R., Liu P., Huang P. (2019). Synergy between auranofin and celecoxib against colon cancer in vitro and in vivo through a novel redox-mediated mechanism. Cancers.

[12] Mao J.T., Roth M.D., Fishbein M.C., Aberle D.R., Zhang Z.F., Rao J.Y., Tashkin D.P., Goodglick L., Holmes E.C., Cameron R.B. (2011). Lung cancer chemoprevention with celecoxib in former smokers. Cancer Prev. Res..

[13] Sabichi A.L., Lee J.J., Grossman H.B., Liu S., Richmond E., Czerniak B.A., De la Cerda J., Eagle C., Viner J.L., Palmer J.L. (2011). A randomized controlled trial of celecoxib to prevent recurrence of nonmuscle-invasive bladder cancer. Cancer Prev. Res..

[14] Paulson S.K., Vaughn M.B., Jessen S.M., Lawal Y., Gresk C.J., Yan B., Maziasz T.J., Cook C.S., Karim A. (2001). Pharmacokinetics of celecoxib after oral administration in dogs and humans: Effect of food and site of absorption. J. Pharmacol. Exp. Ther..

[15] Auda S.H., Fathalla D., Fetih G., El-Badry M., Shakeel F. (2016). Niosomes as transdermal drug delivery system for celecoxib: In vitro and in vivo studies. Polym. Bull..

[16] Homayouni A., Sadeghi F., Varshosaz J., Garekani H.A., Nokhodchi A. (2014). Comparing various techniques to produce micro/nanoparticles for enhancing the dissolution of celecoxib containing PVP. Eur. J. Pharm. Biopharm..

[17] Salem H.F., Kharshoum R.M., Sayed O.M., Abdel Hakim L.F. (2018). Formulation design and optimization of novel soft glycerosomes for enhanced topical delivery of celecoxib and cupferron by Box–Behnken statistical design. Drug Dev. Ind. Pharm..

[18] Solomon S.D., McMurray J.J., Pfeffer M.A., Wittes J., Fowler R., Finn P., Anderson W.F., Zauber A., Hawk E., Bertagnolli M. (2005). Cardiovascular risk associated with celecoxib in a clinical trial for colorectal adenoma prevention. N. Engl. J. Med..

[19] Ascenso A., Raposo S., Batista C., Cardoso P., Mendes T., Praça F.G., Bentley M.V.L.B., Simões S. (2015). Development, characterization, and skin delivery studies of related ultradeformable vesicles: Transfersomes, ethosomes, and transethosomes. Int. J. Nanomed..

[20] Tawfeek H.M., Abdellatif A.A., Abdel-Aleem J.A., Hassan Y.A., Fathalla D. (2020). Transfersomal gel nanocarriers for enhancement the permeation of lornoxicam. J. Drug Deliv. Sci. Technol..

[21] Abdellatif A.A., Tawfeek H.M. (2016). Transfersomal nanoparticles for enhanced transdermal delivery of clindamycin. Aaps Pharmscitech.

[22] Garg V., Singh H., Bimbrawh S., Kumar Singh S., Gulati M., Vaidya Y., Kaur P. (2017). Ethosomes and transfersomes: Principles, perspectives and practices. Curr. Drug Deliv..

[23] Bragagni M., Mennini N., Maestrelli F., Cirri M., Mura P. (2012). Comparative study of liposomes, transfersomes and ethosomes as carriers for improving topical delivery of celecoxib. Drug Deliv..

[24] Cevc G., Blume G. (2001). New, highly efficient formulation of diclofenac for the topical, transdermal administration in ultradeformable drug carriers, Transfersomes. Biochim. Biophys. Acta (BBA)-Biomembr..

[25] Elsayed M.M., Abdallah O., Naggar V., Khalafallah N. (2007). Deformable liposomes and ethosomes as carriers for skin delivery of ketotifen. Die Pharm.-Int. J. Pharm. Sci..

[26] Rattanapak T., Young K., Rades T., Hook S. (2012). Comparative study of liposomes, transfersomes, ethosomes and cubosomes for transcutaneous immunisation: Characterisation and in vitro skin penetration. J. Pharm. Pharmacol..

[27] Faisal W., Soliman G.M., Hamdan A.M. (2018). Enhanced skin deposition and delivery of voriconazole using ethosomal preparations. J. Liposome Res..

[28] Fathalla D., Youssef E.M., Soliman G.M. (2020). Liposomal and ethosomal gels for the topical delivery of anthralin: Preparation, comparative evaluation and clinical assessment in psoriatic patients. Pharmaceutics.

[29] Fathalla D., Soliman G., Fouad E. (2015). Development and in vitro/in vivo evaluation of liposomal gels for the sustained ocular delivery of latanoprost. J Clin Exp Ophthalmol.

[30] Dholakia M., Thakkar V., Patel N., Gandhi T. (2012). Development and characterisation of thermo reversible mucoadhesive moxifloxacin hydrochloride in situ ophthalmic gel. J. Pharm. Bioallied Sci..

[31] Allam A., Elsabahy M., El Badry M., Eleraky N.E. (2021). Betaxolol-loaded niosomes integrated within pH-sensitive in situ forming gel for management of glaucoma. Int. J. Pharm..

[32] Mircioiu C., Voicu V., Anuta V., Tudose A., Celia C., Paolino D., Fresta M., Sandulovici R., Mircioiu I. (2019). Mathematical modeling of release kinetics from supramolecular drug delivery systems. Pharmaceutics.

[33] Ritger P.L., Peppas N.A. (1987). A simple equation for description of solute release II. Fickian and anomalous release from swellable devices. J. Control. Release.

[34] Korsmeyer R.W., Gurny R., Doelker E., Buri P., Peppas N.A. (1983). Mechanisms of solute release from porous hydrophilic polymers. Int. J. Pharm..

[35] Hixson A., Crowell J. (1931). Dependence of reaction velocity upon surface and agitation. Ind. Eng. Chem..

[36] Baker R. (1974). Controlled release: Mechanisms and rates. Control. Release Biol. Act. Agents..

[37] Jain S., Tiwary A.K., Sapra B., Jain N.K. (2007). Formulation and evaluation of ethosomes for transdermal delivery of lamivudine. Aaps Pharmscitech.

[38] Garber J.C., Wayne Barbee R., Bielitzki J.T., Clayton L.A., Donovan J.C., Hendriksen C., Kohn D.F., Lipman N.S., Locke P.A., Melcher J. (2011). Committee for the Update of the Guide for the Care and Use of Laboratory Animals. Guide for the Care and Use of Laboratory Animals.

[39] Sloan K.B., Beall H.D., Weimar W.R., Villanueva R. (1991). The effect of receptor phase composition on the permeability of hairless mouse skin in diffusion cell experiments. Int. J. Pharm..

[40] Mosmann T. (1983). Rapid colorimetric assay for cellular growth and survival: Application to proliferation and cytotoxicity assays. J. Immunol. Methods.

[41] Hamed M.A., Aboul Naser A.F., Aboutabl M.E., Osman A.F., Hassan E.E., Aziz W.M., Khalil W.K., Farghaly A.A., El-Hagrassi A.M. (2021). Bioactive compounds and therapeutic role of *Brassica oleracea* L. seeds in rheumatoid arthritis rats via regulating inflammatory signalling pathways and antagonizing interleukin-1 receptor action. Biomarkers.

[42] Salem N.A., Wahba M.A., Eisa W.H., El-Shamarka M., Khalil W. (2018). Silver oxide nanoparticles alleviate indomethacin-induced gastric injury: A novel antiulcer agent. Inflammopharmacology.

[43] Hamza A.H., Abdulfattah H.M., Mahmoud R.H., Khalil W.K., Ahmed H.H. (2015). Current concepts in pathophysiology and management of hepatocellular carcinoma. Acta Biochim. Pol..

[44] Elhinnawi M.A., Mohareb R.M., Rady H.M., Khalil W.K., Abd Elhalim M.M., Elmegeed G.A. (2018). Novel pregnenolone derivatives modulate apoptosis via Bcl-2 family genes in hepatocellular carcinoma in vitro. J. Steroid Biochem. Mol. Biol..

[45] Krähn G., Leiter U., Udart M., Kaskel P., Peter R. (2001). UVB-induced Decrease of p16/CDKN2A Expression in Skin Cancer Patients. Pigment Cell Res..

[46] Pacifico A., Goldberg L.H., Peris K., Chimenti S., Leone G., Ananthaswamy H. (2008). Loss of CDKN2A and p14ARF expression occurs frequently in human nonmelanoma skin cancers. Br. J. Dermatol..

[47] Patel S., Wilkinson C.J., Sviderskaya E.V. (2020). Loss of both CDKN2A and CDKN2B allows for centrosome overduplication in melanoma. J. Investig. Dermatol..

[48] Olive P.L., Banáth J.P., Durand R.E. (2012). Heterogeneity in radiation-induced DNA damage and repair in tumor and normal cells measured using the “comet” assay. Radiat. Res..

[49] Collins A., Dušinská M., Franklin M., Somorovská M., Petrovská H., Duthie S., Fillion L., Panayiotidis M., Rašlová K., Vaughan N. (1997). Comet assay in human biomonitoring studies: Reliability, validation, and applications. Environ. Mol. Mutagen..

[50] Yawata A., Adachi M., Okuda H., Naishiro Y., Takamura T., Hareyama M., Takayama S., Reed J.C., Imai K. (1998). Prolonged cell survival enhances peritoneal dissemination of gastric cancer cells. Oncogene.

[51] Abdulbaqi I.M., Darwis Y., Khan N.A.K., Abou Assi R., Khan A.A. (2016). Ethosomal nanocarriers: The impact of constituents and formulation techniques on ethosomal properties, in vivo studies, and clinical trials. Int. J. Nanomed..

[52] El Zaafarany G.M., Awad G.A., Holayel S.M., Mortada N.D. (2010). Role of edge activators and surface charge in developing ultradeformable vesicles with enhanced skin delivery. Int. J. Pharm..

[53] Albash R., Abdelbary A.A., Refai H., El-Nabarawi M.A. (2019). Use of transethosomes for enhancing the transdermal delivery of olmesartan medoxomil: In vitro, ex vivo, and in vivo evaluation. Int. J. Nanomed..

[54] Aboud H.M., Ali A.A., El-Menshawe S.F., Elbary A.A. (2016). Nanotransfersomes of carvedilol for intranasal delivery: Formulation, characterization and in vivo evaluation. Drug Deliv..

[55] Kunieda H., Ohyama K.-I. (1990). Three-phase behavior and HLB numbers of bile salts and lecithin in a water-oil system. J. Colloid Interface Sci..

[56] Samuel G., Nazim U., Sharma A., Manuel V., Elnaggar M.G., Taye A., Nasr N.E.H., Hofni A., Hakiem A.F.A. (2022). Selective Targeting of the Novel CK-10 Nanoparticles to the MDA-MB-231 Breast Cancer Cells. J. Pharm. Sci..

[57] Yusuf M., Sharma V., Pathak K. (2014). Nanovesicles for transdermal delivery of felodipine: Development, characterization, and pharmacokinetics. Int. J. Pharm. Investig..

[58] Khalil R.M., Abdelbary G.A., Basha M., Awad G.E., El-Hashemy H.A. (2017). Enhancement of lomefloxacin Hcl ocular efficacy via niosomal encapsulation: In vitro characterization and in vivo evaluation. J. Liposome Res..

[59] Mbah C.C., Builders P.F., Attama A.A. (2014). Nanovesicular carriers as alternative drug delivery systems: Ethosomes in focus. Expert Opin. Drug Deliv..

[60] Ascenso A., Cruz M., Euletério C., Carvalho F.A., Santos N.C., Marques H.C., Simoes S. (2013). Novel tretinoin formulations: A drug-in-cyclodextrin-in-liposome approach. J. Liposome Res..

[61] Junyaprasert V.B., Singhsa P., Suksiriworapong J., Chantasart D. (2012). Physicochemical properties and skin permeation of Span 60/Tween 60 niosomes of ellagic acid. Int. J. Pharm..

[62] Mokhtar M., Sammour O.A., Hammad M.A., Megrab N.A. (2008). Effect of some formulation parameters on flurbiprofen encapsulation and release rates of niosomes prepared from proniosomes. Int. J. Pharm..

[63] Hao Y., Zhao F., Li N., Yang Y. (2002). Studies on a high encapsulation of colchicine by a niosome system. Int. J. Pharm..

[64] Alajami H.N., Fouad E.A., Ashour A.E., Kumar A., Yassin A.E.B. (2022). Celecoxib-Loaded Solid Lipid Nanoparticles for Colon Delivery: Formulation Optimization and In Vitro Assessment of Anti-Cancer Activity. Pharmaceutics.

[65] Suzuki H., Ogawa M., Hironaka K., Ito K., Sunada H. (2001). A nifedipine coground mixture with sodium deoxycholate. II. Dissolution characteristics and stability. Drug Dev. Ind. Pharm..

[66] Pandya V.M., Patel D.J., Patel J.K., Patel R.P. (2009). Formulation, characterization, and optimization of fast-dissolve tablets containing celecoxib solid dispersion. Dissolution Technol..

[67] Salama A., Badran M., Elmowafy M., Soliman G.M. (2019). Spironolactone-loaded leciplexes as potential topical delivery systems for female acne: In vitro appraisal and ex vivo skin permeability studies. Pharmaceutics.

[68] Sun S., Liang N., Kawashima Y., Xia D., Cui F. (2011). Hydrophobic ion pairing of an insulin-sodium deoxycholate complex for oral delivery of insulin. Int. J. Nanomed..

[69] Eleraky N.E., Omar M.M., Mahmoud H.A., Abou-Taleb H.A. (2020). Nanostructured lipid carriers to mediate brain delivery of temazepam: Design and in vivo study. Pharmaceutics.

[70] Badria F., Mazyed E. (2020). Formulation of Nanospanlastics as a Promising Approach for Improving the Topical Delivery of a Natural Leukotriene Inhibitor (3-Acetyl-11-Keto-β-Boswellic Acid): Statistical Optimization, in vitro Characterization, and ex vivo Permeation Study. Drug Des. Dev. Ther..

[71] Ana R., Mendes M., Sousa J., Pais A., Falcão A., Fortuna A., Vitorino C. (2019). Rethinking carbamazepine oral delivery using polymer-lipid hybrid nanoparticles. Int. J. Pharm..

[72] Elnaggar M.G., Jiang K., Eldesouky H.E., Pei Y., Park J., Yuk S.A., Meng F., Dieterly A.M., Mohammad H.T., Hegazy Y.A. (2020). Antibacterial nanotruffles for treatment of intracellular bacterial infection. Biomaterials.

[73] Fissan H., Ristig S., Kaminski H., Asbach C., Epple M. (2014). Comparison of different characterization methods for nanoparticle dispersions before and after aerosolization. Anal. Methods.

[74] Abdellatif A.A., Rasheed Z., Alhowail A.H., Alqasoumi A., Alsharidah M., Khan R.A., Aljohani A.S., Aldubayan M.A., Faisal W. (2020). Silver citrate nanoparticles inhibit PMA-induced TNFα expression via deactivation of NF-κB activity in human cancer cell-lines, MCF-7. Int. J. Nanomed..

[75] Mekkawy A.I., Eleraky N.E., Soliman G.M., Elnaggar M.G., Elnaggar M.G. (2022). Combinatorial Therapy of Letrozole-and Quercetin-Loaded Spanlastics for Enhanced Cytotoxicity against MCF-7 Breast Cancer Cells. Pharmaceutics.

[76] El-Sayed M.M., Hussein A.K., Sarhan H.A., Mansour H.F. (2017). Flurbiprofen-loaded niosomes-in-gel system improves the ocular bioavailability of flurbiprofen in the aqueous humor. Drug Dev. Ind. Pharm..

[77] Mohanty D., Rani M.J., Haque M.A., Bakshi V., Jahangir M.A., Imam S.S., Gilani S.J. (2020). Preparation and evaluation of transdermal naproxen niosomes: Formulation optimization to preclinical anti-inflammatory assessment on murine model. J. Liposome Res..

[78] Kaul S., Jain N., Nagaich U. (2022). Ultra deformable vesicles for boosting transdermal delivery of 2-arylpropionic acid class drug for management of musculoskeletal pain. J. Pharm. Investig..

[79] Faria M.J., Machado R., Ribeiro A., Gonçalves H., Real Oliveira M.E.C., Viseu T., das Neves J., Lúcio M. (2019). Rational development of liposomal hydrogels: A strategy for topical vaginal antiretroviral drug delivery in the context of HIV prevention. Pharmaceutics.

[80] Ricci E., Lunardi L.O., Nanclares D., Marchetti J.M. (2005). Sustained release of lidocaine from Poloxamer 407 gels. Int. J. Pharm..

[81] El-Badry M., Fetih G. (2011). Preparation, charactarization and anti-inflammatory activity of celecoxib chitosan gel formulations. J. Drug Deliv. Sci. Technol..

[82] Baig M.S., Ahad A., Aslam M., Imam S.S., Aqil M., Ali A. (2016). Application of Box–Behnken design for preparation of levofloxacin-loaded stearic acid solid lipid nanoparticles for ocular delivery: Optimization, in vitro release, ocular tolerance, and antibacterial activity. Int. J. Biol. Macromol..

[83] Allam A.A., Eleraky N.E., Diab N.H., Elsabahy M., Mohamed S.A., Abdel-Ghaffar H.S., Hassan N.A., Shouman S.A., Omran M.M., Hassan S.B. (2022). Development of Sedative Dexmedetomidine Sublingual In Situ Gels: In Vitro and In Vivo Evaluations. Pharmaceutics.

[84] Salem H.F., Kharshoum R.M., Abou-Taleb H.A., AbouTaleb H.A., AbouElhassan K.M. (2019). Progesterone-loaded nanosized transethosomes for vaginal permeation enhancement: Formulation, statistical optimization, and clinical evaluation in anovulatory polycystic ovary syndrome. J. Liposome Res..

[85] Salem H.F., Nafady M.M., Kharshoum R.M., Abd el-Ghafar O.A., Farouk H.O. (2020). Novel Enhanced Therapeutic Efficacy of Dapoxetine HCl by Nano-Vesicle Transdermal Gel for Treatment of Carrageenan-Induced Rat Paw Edema. AAPS PharmSciTech.

[86] Barry B.W. (2001). Novel mechanisms and devices to enable successful transdermal drug delivery. Eur. J. Pharm. Sci..

[87] Mishra K.K., Kaur C.D., Verma S., Sahu A.K., Dash D.K., Kashyap P., Mishra S.P. (2019). Transethosomes and nanoethosomes: Recent approach on transdermal drug delivery system. Nanomedicine.

[88] Shaji J., Bajaj R. (2018). Transethosomes: A new prospect for enhanced transdermal delivery. Int. J. Pharm. Sci. Res..

[89] Ferrara F., Benedusi M., Sguizzato M., Cortesi R., Baldisserotto A., Buzzi R., Valacchi G., Esposito E. (2022). Ethosomes and Transethosomes as Cutaneous Delivery Systems for Quercetin: A Preliminary Study on Melanoma Cells. Pharmaceutics.

[90] Rosas C., Sinning M., Ferreira A., Fuenzalida M., Lemus D. (2014). Celecoxib decreases growth and angiogenesis and promotes apoptosis in a tumor cell line resistant to chemotherapy. Biol. Res..

[91] Perumal V., Banerjee S., Das S., Sen R.K., Mandal M. (2011). Effect of liposomal celecoxib on proliferation of colon cancer cell and inhibition of DMBA-induced tumor in rat model. Cancer Nanotechnol..

[92] Whiteman D.C., Green A.C. (1999). Melanoma and sun exposure: Where are we now?. Int. J. Dermatol..

[93] Florell S.R., Boucher K.M., Garibotti G., Astle J., Kerber R., Mineau G., Wiggins C., Noyes R.D., Tsodikov A., Cannon-Albright L.A. (2005). Population-based analysis of prognostic factors and survival in familial melanoma. J. Clin. Oncol..

[94] Goldstein A.M., Chan M., Harland M., Hayward N.K., Demenais F., Bishop D.T., Azizi E., Bergman W., Bianchi-Scarra G., Bruno W. (2007). Features associated with germline CDKN2A mutations: A GenoMEL study of melanoma-prone families from three continents. J. Med. Genet..

[95] Su C.-C., Wang M.-J., Chiu T.-L. (2010). The anti-cancer efficacy of curcumin scrutinized through core signaling pathways in glioblastoma. Int. J. Mol. Med..

[96] Liu W., Chen Y., Wang W., Keng P., Finkelstein J., Hu D., Liang L., Guo M., Fenton B., Okunieff P. (2003). Combination of radiation and celebrex (celecoxib) reduce mammary and lung tumor growth. Am. J. Clin. Oncol..

[97] Kang K.B., Zhu C., Yong S.K., Gao Q., Wong M.C. (2009). Enhanced sensitivity of celecoxib in human glioblastoma cells: Induction of DNA damage leading to p53-dependent G1 cell cycle arrest and autophagy. Mol. Cancer.

[98] Kalantar M., Rezaei M., Moghimipour E., Bavarsad N., Kalantari H., Varnaseri G., Forouzan A. (2015). Evaluation of Apoptosis Induced by Celecoxib Loaded Liposomes in Isolated Rat Hepatocytes. Jundishapur J. Nat. Pharm. Prod..

[99] Pritchard R., Rodríguez-Enríquez S., Pacheco-Velázquez S.C., Bortnik V., Moreno-Sánchez R., Ralph S. (2018). Celecoxib inhibits mitochondrial O2 consumption, promoting ROS dependent death of murine and human metastatic cancer cells via the apoptotic signalling pathway. Biochem. Pharmacol..

[100] Ansari K.M., Rundhaug J.E., Fischer S.M. (2008). Multiple Signaling Pathways Are Responsible for Prostaglandin E2–Induced Murine Keratinocyte Proliferation. Mol. Cancer Res..

[101] Rundhaug J.E., Pavone A., Kim E., Fischer S.M. (2007). The effect of cyclooxygenase-2 overexpression on skin carcinogenesis is context dependent. Mol. Carcinog. Publ. Coop. Univ. Tex. MD Cancer Cent..

[102] Tjiu J.-W., Chen J.-S., Shun C.-T., Lin S.-J., Liao Y.-H., Chu C.-Y., Tsai T.-F., Chiu H.-C., Dai Y.-S., Inoue H. (2009). Tumor-associated macrophage-induced invasion and angiogenesis of human basal cell carcinoma cells by cyclooxygenase-2 induction. J. Investig. Dermatol..

[103] Elmets C.A., Viner J.L., Pentland A.P., Cantrell W., Lin H.-Y., Bailey H., Kang S., Linden K.G., Heffernan M., Duvic M. (2010). Chemoprevention of nonmelanoma skin cancer with celecoxib: A randomized, double-blind, placebo-controlled trial. J. Natl. Cancer Inst..

[104] Shureiqi I., Chen D., Lotan R., Yang P., Newman R.A., Fischer S.M., Lippman S.M. (2000). 15-Lipoxygenase-1 mediates nonsteroidal anti-inflammatory drug-induced apoptosis independently of cyclooxygenase-2 in colon cancer cells. Cancer Res..

[105] Vaish V., Sanyal S.N. (2012). Role of Sulindac and Celecoxib in chemoprevention of colorectal cancer via intrinsic pathway of apoptosis: Exploring NHE-1, intracellular calcium homeostasis and Calpain 9. Biomed. Pharmacother..

[106] Piplani H., Rana C., Vaish V., Vaiphei K., Sanyal S. (2013). Dolastatin, along with Celecoxib, stimulates apoptosis by a mechanism involving oxidative stress, membrane potential change and PI3-K/AKT pathway down regulation. Biochim. Biophys. Acta (BBA)-Gen. Subj..

[107] Ramer R., Walther U., Borchert P., Laufer S., Linnebacher M., Hinz B. (2013). Induction but not inhibition of COX-2 confers human lung cancer cell apoptosis by celecoxib. J. Lipid Res..

[108] Liu X., Yue P., Zhou Z., Khuri F.R., Sun S.-Y. (2004). Death receptor regulation and celecoxib-induced apoptosis in human lung cancer cells. J. Natl. Cancer Inst..

