# Supplementary material for: Transethosomal Gel for the Topical Delivery of Celecoxib: Formulation and Estimation of Skin Cancer Progression
Source: Pharmaceutics. 2022 Dec 21;15(1):22. doi: 10.3390/pharmaceutics15010022 (PMC9864437; doi:10.3390/pharmaceutics15010022)
Supplement: Supplementary file 1 [file pharmaceutics-15-00022-s001.zip › pharmaceutics-2062337-supplementary.pdf]

Table S1: Kinetic analysis for release of CXB-TES- carbopol 934 hydrogel.

	zero	First	Diffusion	Peppas	
a	4.464798	1.981193	-1.50307	0.850337	
b	0.933486	-0.00476	5.476695	2.253622	chosen "r"
r	0.97343	-0.98105	0.998015	0.853522	0.99801
k	0.933486	-0.01097	5.476695	2.253622	
t(1/2)	53.56266	-63.1547	83.34948		

Table S2: Kinetic analysis for release of CXB-TES- HPMC hydrogel.

	zero	First	Diffusion	Peppas	
a	6.833925	1.970335	-0.13182	0.850337	
b	1.042397	-0.00552	6.252248	2.253622	chosen "r"
r	0.94881	-0.96237	0.994499	0.853522	0.994499
k	1.042397	-0.01272	6.252248	2.253622	
t(1/2)	47.96635	-54.4955	63.95398		

Table S3: Korsmeyer-Peppas Kinetic analysis for release of CXB-TES- HPMC hydrogel.

**0.5**	**3.21**		0.506730618	-0.30103
**1**	5.41		0.73325471	0
**2**	9.01		0.954960288	0.30103
**3**	11.12		1.04595436	0.477121
**4**	13.35		1.125602814	0.60206
**6**	15.33		1.185559517	0.778151
**12**	22.70		1.356113896	1.079181
**24**	29.25		1.466198515	1.380211
		**n**	**0.568018281**	
		**r**	**0.991086837**	

Table S4: Korsmeyer-Peppas Kinetic analysis for release of CXB-TES- carbapol hydrogel.

**0.5**	**2.67**		0.426402661	-0.30103
**1**	3.90		0.591433759	0
**2**	6.20		0.792141842	0.30103
**3**	7.57		0.878899173	0.477121
**4**	9.83		0.99250386	0.60206
**6**	11.20		1.049179786	0.778151
**12**	18.27		1.261646219	1.079181
**24**	25.10		1.399617327	1.380211
		**n**	**0.589525475**	
		**r**	**0.998385892**	

Table S5: Visual score of DNA damage in normal skin cell lines treated with different CXB preparations.

Treatment	No. of cells			Class**				DNA damaged cells % (Mean±SEM)
	No of samples	Analyzed*	Comets	0	1	2	3	
**Normal cells (-ve)**	5	500	34	466	29	5	0	6.82±0.86[c]
**(-ve)+TES suspension**	5	500	93	407	32	25	36	18.84±1.28[b]
**(-ve)+Blank HPMC gel**	5	500	38	462	30	6	2	7.61±0.51[c]
**(-ve)+CXB gel**	5	500	46	454	28	11	7	9.22±0.58[c]
**(-ve)+CXB-TES gel**	5	500	87	413	31	23	33	17.44±1.03[b]
**(-ve)+ CXB powder**	5	500	118	382	37	33	48	23.63±0.93[a]
**Normal cells + Doxorubicin (+ve)**	5	500	107	393	34	29	44	21.41±0.75[ab]

Table S6: Visual score of DNA damage in skin cancer cell lines treated with different CXB preparations.

Treatment	No. of cells			Class**				DNA damaged cells % (Mean±SEM)
	No of samples	Analyzed*	Comets	0	1	2	3	
**Skin cancer (-ve)**	5	500	56	444	28	13	15	11.21±0.87[d]
**(-ve)+TES suspension**	5	500	117	383	39	33	45	23.43±0.93[ab]
**(-ve)+Blank HPMC gel**	5	500	69	431	31	17	21	13.82±0.66[cd]
**(-ve)+CXB gel**	5	500	81	419	35	20	26	16.23±1.07[c]
**(-ve)+CXB-TES gel**	5	500	107	393	34	31	42	21.41±0.51[b]
**(-ve)+CXB powder**	5	500	138	362	33	42	63	27.63±1.21[a]
**Skin cancer + Doxorubicin (+ve)**	5	500	124	376	32	38	54	24.82±0.80[ab]

*: Number of cells examined per a group, **: Class 0= no tail; 1= tail length < diameter of nucleus; 2= tail length between 1X and 2X the diameter of nucleus; and 3= tail length > 2X the diameter of nucleus. Data are presented as mean ± SD. [a,b,c:] Mean values within tissue with unlike superscript letters were significantly different ($p \leq 0.05$; ANOVA/Tukey).

Table S7: DNA fragmentation detected in normal skin cell lines.

Treatment	DNA Fragmentation (%) Mean ± SEM
**Normal cells (-ve)**	**8.1±0.24[d]**
**(-ve)+TES suspension**	**20.6±0.56[b]**
**(-ve)+Blank HPMC gel**	**15.2±0.30[c]**
**(-ve)+CXB gel**	**17.1±0.23[bc]**
**(-ve)+CXB-TES gel**	**19.4±0.48[b]**
**(-ve)+ CXB powder**	**27.6±0.54[a]**
**Normal cells + Doxorubicin (+ve)**	**21.7±0.66[b]**

Means with different superscripts ([a, b, c, d]) between treatments in the same column are significantly different at $p \leq 0.05$; ANOVA/Tukey.

Table S8: DNA fragmentation detected in cancer skin cell lines.

Treatment	DNA Fragmentation (%) Mean ± SEM
**Skin cancer (-ve)**	**13.7±0.45[f]**
**(-ve)+TES suspension**	**35.5±0.58[b]**
**(-ve)+Blank HPMC gel**	**17.9±0.15[e]**
**(-ve)+CXB gel**	**21.2±0.66[d]**
**(-ve)+CXB-TES gel**	**31.6±0.61[c]**
**(-ve)+CXB powder**	**43.7±0.53[a]**
**Skin cancer + Doxorubicin (+ve)**	**33.8±0.48[bc]**

Means with different superscripts ([a, b, c, d, e, f]) between treatments in the same column are significantly different at $p \leq 0.05$; ANOVA/Tukey.
